# Expanding the triangle of U: comparative analysis of the *Hirschfeldia incana* genome provides insights into chromosomal evolution, phylogenomics and high photosynthesis-related traits

**DOI:** 10.1093/aob/mcae179

**Published:** 2024-10-24

**Authors:** Nam V Hoang, Nora Walden, Ludovico Caracciolo, Sofia Bengoa Luoni, Moges Retta, Run Li, Felicia C Wolters, Tina Woldu, Frank F M Becker, Patrick Verbaarschot, Jeremy Harbinson, Steven M Driever, Paul C Struik, Herbert van Amerongen, Dick de Ridder, Mark G M Aarts, M Eric Schranz

**Affiliations:** Biosystematics Group, Wageningen University and Research, Droevendaalsesteeg 1, 6708 PB Wageningen, The Netherlands; Centre for Organismal Studies, Heidelberg University, 69120 Heidelberg, Germany; Laboratory of Biophysics, Wageningen University and Research, Stippeneng 4, 6708 WE Wageningen, The Netherlands; Laboratory of Genetics, Wageningen University and Research, Droevendaalsesteeg 1, 6708 PB Wageningen, The Netherlands; Centre for Crop Systems Analysis, Wageningen University and Research, PO Box 430, 6700 AK Wageningen, The Netherlands; Biosystematics Group, Wageningen University and Research, Droevendaalsesteeg 1, 6708 PB Wageningen, The Netherlands; Biosystematics Group, Wageningen University and Research, Droevendaalsesteeg 1, 6708 PB Wageningen, The Netherlands; Bioinformatics Group, Wageningen University and Research, Droevendaalsesteeg 1, 6708 PB Wageningen, The Netherlands; Bioinformatics Group, Wageningen University and Research, Droevendaalsesteeg 1, 6708 PB Wageningen, The Netherlands; Laboratory of Genetics, Wageningen University and Research, Droevendaalsesteeg 1, 6708 PB Wageningen, The Netherlands; Biosystematics Group, Wageningen University and Research, Droevendaalsesteeg 1, 6708 PB Wageningen, The Netherlands; Laboratory of Biophysics, Wageningen University and Research, Stippeneng 4, 6708 WE Wageningen, The Netherlands; Centre for Crop Systems Analysis, Wageningen University and Research, PO Box 430, 6700 AK Wageningen, The Netherlands; Centre for Crop Systems Analysis, Wageningen University and Research, PO Box 430, 6700 AK Wageningen, The Netherlands; Laboratory of Biophysics, Wageningen University and Research, Stippeneng 4, 6708 WE Wageningen, The Netherlands; Bioinformatics Group, Wageningen University and Research, Droevendaalsesteeg 1, 6708 PB Wageningen, The Netherlands; Laboratory of Genetics, Wageningen University and Research, Droevendaalsesteeg 1, 6708 PB Wageningen, The Netherlands; Biosystematics Group, Wageningen University and Research, Droevendaalsesteeg 1, 6708 PB Wageningen, The Netherlands

**Keywords:** *Hirschfeldia incana*, Brassicaceae, Brassiceae, Brassica U triangle, hybridization origin, whole-genome duplication, photosynthesis evolution, polyploidy, sub-genome dominance

## Abstract

**Background and Aims:**

The Brassiceae tribe encompasses many economically important crops and exhibits high intra- and interspecific phenotypic variation. After a shared whole-genome triplication (WGT) event (*Br-α*, ~15.9 Mya), differential lineage diversification and genomic changes contributed to an array of divergence in morphology, biochemistry and physiology underlying photosynthesis-related traits. Here, the C_3_ species *Hirschfeldia incana* is studied because it displays high photosynthetic rates in high-light conditions. Our aim was to elucidate the evolution that gave rise to the genome of *H. incana* and its high-photosynthesis traits.

**Methods:**

We reconstructed a chromosome-level genome assembly for *H. incana* (Nijmegen, v.2.0) using nanopore and chromosome conformation capture (Hi-C) technologies, with 409 Mb in size and an N50 of 52 Mb (a 10× improvement over the previously published scaffold-level v.1.0 assembly). The updated assembly and annotation were subsequently used to investigate the WGT history of *H. incana* in a comparative phylogenomic framework from the Brassiceae ancestral genomic blocks and related diploidized crops.

**Key Results:**

*Hirschfeldia incana* (*x* = 7) shares extensive genome collinearity with *Raphanus sativus* (*x* = 9). These two species share some commonalities with *Brassica rapa* and *Brassica oleracea* (A genome, *x* = 10 and C genome, *x* = 9, respectively) and other similarities with *Brassica nigra* (B genome, *x* = 8). Phylogenetic analysis revealed that *H. incana* and *R. sativus* form a monophyletic clade in between the *Brassica* A/C and B genomes. We postulate that *H. incana* and *R. sativus* genomes are results of hybridization or introgression of the *Brassica* A/C and B genome types. Our results might explain the discrepancy observed in published studies regarding phylogenetic placement of *H. incana* and *R. sativus* in relationship to the ‘triangle of U’ species. Expression analysis of WGT retained gene copies revealed sub-genome expression divergence, probably attributable to neo- or sub-functionalization. Finally, we highlight genes associated with physio-biochemical–anatomical adaptive changes observed in *H. incana*, which are likely to facilitate its high-photosynthesis traits under high light.

**Conclusions:**

The improved *H. incana* genome assembly, annotation and results presented in this work will be a valuable resource for future research to unravel the genetic basis of its ability to maintain a high photosynthetic efficiency in high-light conditions and thereby improve photosynthesis for enhanced agricultural production.

## INTRODUCTION

The Brassicaceae family contains the model plant *Arabidopsis thaliana* and many economically important vegetable, root and oil crops. Many of these crops are members of the Brassiceae tribe (*Brassicas*) that underwent a meso-hexaploidy *Brassica α* whole-genome triplication event (*Br-α* WGT), which occurred ~15.9 Mya in the middle of the Miocene epoch (Jiao *et al.*, 2011; Liu *et al.*, 2014). This has resulted in a massive inter- and intraspecific phenotypic variation owing to differential chromosomal rearrangements (i.e. diploidization) and gene retention and loss (i.e. fractionation) (Cheng *et al.*, 2014, 2016).

After the *Br-α* WGT event, differential lineage diversification and genomic changes among the Brassiceae species contributed to an array of divergence in morphology, biochemistry and physiology underlying photosynthesis-related traits (Guerreiro *et al.*, 2023; Schluter *et al.*, 2023). Although no true C_4_ photosynthesis in Brassiceae species has been reported so far, the tribe consists of species that exhibit a wide range of light-saturated photosynthetic rates and use both the C_3_ and C_3_–C_4_ photosynthetic pathways (Schluter *et al.*, 2023). Among these, the C_3_ species *Hirschfeldia incana* (grey mustard, *n* = *x* = 7) was reported to display high photosynthetic rates (i.e. carbon assimilation) in high-light conditions (Canvin *et al.*, 1980; Garassino *et al.*, 2022). This, together with its close evolutionary proximity to the model plant *Arabidopsis* and to *Brassica* crops, positions *H. incana* as a good model to study how high-photosynthesis traits have evolved in the Brassiceae tribe.

In the last two decades, significant genomic resources were developed for *Arabidopsis* and *Brassica* crops, including *Brassica rapa*, *Brassica oleracea*, *Brassica nigra* and their allo-tetraploid hybrids which are part of the ‘triangle of U’ (Wu *et al.*, 2022). However, genomic data available for *H. incana* and its wild relatives in the Brassiceae tribe are still limited, which hinders our understanding of the genetic basis of its high photosynthesis in high-light conditions. More recently, high-quality chromosome-level genome sequences of the close relatives *Raphanus sativus* and *Sinapis arvensis* were released (Cho *et al.*, 2022; Xu *et al.*, 2023; Yang *et al.*, 2023). Notably, a new genomic panel was developed for a total of 18 Brassiceae species at scaffold level with different photosynthesis types, including C_3_ and C_3_–C_4_ (Guerreiro *et al.*, 2023). These resources are expected to support large-scale comparative phylogenomic studies in combination with high-throughput phenotyping, focusing on the *Nigra–Rapa/Oleracea–Raphanus* clades to untangle the evolution of the high-photosynthesis traits in the Brassiceae tribe. To date, there are at least two scaffold-level genome assemblies of different *H. incana* accessions [Nijmegen (NIJ) and HIR1] (Garassino *et al.*, 2022; Guerreiro *et al.*, 2023) and a few transcriptome datasets (Mabry *et al.*, 2020; Garassino *et al.*, 2022, 2024; Hasnaoui *et al.*, 2022), which have facilitated studies on comparative genomics and the expression of genes related to photosynthesis and lead resistance traits. However, for genome evolution and synteny-based studies, it is imperative to have chromosome-level assemblies to uncover the evolutionary trajectory that led to these interesting and important traits in this species. This is because genome evolution and synteny, i.e. conserved gene order across different genomes (Liu *et al.*, 2018), allow the detection of chromosome-level reorganization events, including whole-genome duplication/triplication (WGD/WGT), that gave rise to different evolutionary groups.

It has been proposed that the Brassiceae genomes underwent a ‘two-step’ hybridization (Cheng *et al.*, 2012, 2014; Hao *et al.*, 2021) that resulted in three distinct sub-genomes of different origins, namely the least-fractionated (LF), medium-fractionated (MF_1_) and most-fractionated (MF_2_) sub-genomes. During this process, the MF_1_ and MF_2_ sub-genomes initially emerged together through an auto-tetraploidization event, followed by a first round of diploidization and fractionation. The LF sub-genome was added subsequently to form an allo-hexaploid genome, which was also followed by another round of diploidization and fractionation. Pioneering work suggested that these sub-genomes were derived from the common ancestral tPCK (translocated proto-Calepineae karyotype) of all Brassiceae species (Schranz *et al.*, 2006; Lysak *et al.*, 2016). Typically, the three sub-genomes display differential gene fractionation rates and a gene expression bias, with the LF sub-genome being dominant. From a broader perspective, this sub-genome dominance phenomenon has been observed in several families, including Brassicaceae (Cheng *et al.*, 2012; Liu *et al.*, 2014; Perumal *et al.*, 2020; Yang *et al.*, 2023), Cleomaceae (Hoang *et al.*, 2023), Poaceae (International Wheat Genome Sequencing Consortium, 2014) and Asteraceae (Barker *et al.*, 2016).

Additionally, the tribe Brassiceae, and likewise the Brassicaceae family, is notorious for having phylogenetic trees with poorly supported nodes owing to their complicated history of allo-polyploidization, incomplete lineage sorting and rampant introgressive hybridization (Hendriks *et al.*, 2023). It is also expected that nuclear genes were selected for cyto-nuclear compatibility in the hybrid genotypes, which in turn led to cyto-nuclear phylogenetic discordance (Forsythe *et al.*, 2020). This could mislead our understanding of species relationships and evolution. Regarding the nuclear phylogenetic placement of *H. incana* in relationship to the species in the ‘triangle of U’, published studies appear to be inconsistent. *Hirschfeldia incana* was either grouped closely to *B. rapa*/*B. oleracea* (A/C genome type) by Huang *et al.* (2016) or with *B. nigra* (B genome type) by Garassino *et al.* (2022). Interestingly, this is similar to the case of radish (*R. sativus*) (Cho *et al.*, 2022; Yang *et al.*, 2023). It was previously revealed that the *R. sativus* genome structure exhibits intermediate characteristics between the *Brassica* A/C and B genome types (Jeong *et al.*, 2016; Cho *et al.*, 2022). If this intermediate genome structure is the result of hybridization or introgression, it might explain the observed phylogenetic incongruency among nuclear trees in published studies. For example, different contributions of the progenitor genomes to a set of genetic markers that are used for phylogenetic studies could possibly change the phylogenetic placement of species. With a high-quality genome, it would be possible to investigate the placement of *H. incana* and its relationships with the Brassiceae species through chromosome and genome evolution. This is also crucial for resolving the nuclear genome-based phylogenetic relationships and designation of Brassiceae species, which remain controversial (Huang *et al.*, 2016; Hendriks *et al.*, 2023).

Owing to its importance, photosynthesis has become one of the most studied processes in plant science, with the aim to increase agricultural production. Although the model C_3_ plant *A. thaliana* has been used in most fundamental research and discoveries, several other model systems of different photosynthesis types have also been established, including that of the C_4_ (Brown *et al.*, 2005), C_3_–C_4_ (Gowik *et al.*, 2011) and Crassulacean acid metabolism (CAM) (Edwards, 2019) types. These studies have resulted in the identification of targets for improvement of photosynthesis through manipulation of biochemical metabolic pathways, canopy architecture and leaf anatomy, in addition to the underlying mechanism of natural variation in photosynthesis (Lawson *et al.*, 2012; Tholen *et al.*, 2012; Zhu *et al.*, 2013; Theeuwen *et al.*, 2022). Key genetic factors responsible for these target features would be pivotal to allow redesigning of crops with desirable high-photosynthesis traits. Regarding the ability of *H. incana* to maintain a high photosynthetic efficiency in high-light conditions, recent advances in high-throughput phenotyping and sequencing technologies could facilitate the investigation of its genetic basis and thereby suggest potential targets for photosynthesis improvement. This will also potentially explain the evolution that gave rise to its high-photosynthesis traits.

In this study, we present an improved chromosome-level assembly (v.2.0) of the *H. incana* NIJ accession based on a combination of Oxford Nanopore Technology (ONT) sequencing and chromosome conformation capture (Hi-C) data. We also provide an updated genome annotation that includes more gene models than the previous version and is comparable in gene model number to those of Brassiceae genomes that underwent the *Br-α* WGT event. The improved *H. incana* genome assembly and annotation allowed us to elucidate the genome evolution of this species in relationship to the *Brassica* species and other species within the Brassiceae tribe. We showed that, like the *Brassica* genomes, the *H. incana* genome was also derived from the common ancestral tPCK genomic blocks. The triplicated ancestral genomic blocks within the *H. incana* genome could be classified into three sub-genomes, with the LF sub-genome showing dominance in gene retention and gene expression. The *H. incana* genome appears to be similar to that of *R. sativus* in terms of collinearity and displays intermediate characteristics of *Brassica* A/C and B genome types. The results might explain the discrepancy observed in the published studies regarding the phylogenetic placement of *H. incana* and *R. sativus* in relationship to species within the ‘triangle of U’. Finally, we highlight genes that are associated with the physio-biochemical–anatomical adaptive changes observed in *H. incana* which are likely to facilitate its high rate of carbon assimilation when grown under high light intensity. The updated assembly and annotation will be a valuable resource for future research to explore the genetic basis of this interesting species in terms of retaining a high light-use efficiency under high light intensity; for example, through exploring the natural genetic variations within the *H. incana* accessions or interspecific comparative genomics/transcriptomics to pinpoint the underlying mechanism responsible for its variation in photosynthetic efficiency.

## MATERIALS AND METHODS

### Plant materials

Plant materials used in this study were derived from *H. incana* reference line ‘NIJ’, which was inbred for more than six rounds by hand pollination (i.e. F_6_), as previously described by Garassino *et al.* (2022). For whole-genome and transcriptome sequencing using Nanopore ONT technologies, seeds derived from this line were used. The line was then inbred for another two rounds (i.e. F_8_) in a greenhouse at Wageningen University, The Netherlands, and used for Hi-C sequencing.

### *Whole-genome sequencing of the* H. incana *genome*

For Nanopore ONT sequencing, 750 mg of leaf tissues was collected and used for high-molecular-weight (HMW) genomic DNA extraction according to the LeafGo method (Driguez *et al.*, 2021). An aliquot of 1.2–1.5 μg of HMW genomic DNA was used for library preparation following the Oxford Nanopore SQK-LSK114 kit. We generated three different libraries without size selection and with size selection (two bins of >25 kb and >40 kb thresholds). Each of these libraries was sequenced using a MinION platform on an R10.4.1 flow cell and then combined for downstream analyses. Base calling was performed using Dorado Duplex v.0.3.4 (https://github.com/nanoporetech/dorado) with default parameters and ‘*--min-qscore 10*’ and the ‘*dna_r10.4.1_e8.2_400bps_sup@v4.2.0*’ model to use duplex reads and obtain higher read quality compared with the simplex base-calling method (Supplementary Data Fig. S1).

For Hi-C sequencing based on the Dovetail Omni-C library protocol, chromatin was initially fixed with formaldehyde in the nucleus, then extracted. Extracted chromatin was then digested with DNAse I; chromatin ends were repaired and subsequently ligated to a biotinylated bridge adapter, followed by proximity ligation of adapter-containing ends. After that, crosslinks were reversed, and the DNA was purified. The purified DNA underwent treatment to eliminate any biotin that was not internally bound to ligated fragments. Sequencing libraries were prepared using NEBNext Ultra enzymes and Illumina-compatible adapters. Fragments containing biotin were isolated using streptavidin beads prior to PCR enrichment to achieve the final library. The final library was subsequently sequenced on an Illumina HiSeqX platform to produce ~44× genome coverage (Supplementary Data Fig. S1). All steps were performed by Dovetail Genomics (Scotts Valley, CA, USA).

### *Chromosome-scale assembly of the* H. incana *genome*

The genome size of *H. incana* was re-estimated using a total 102 million Illumina whole-genome sequencing (WGS) data from the study by Garassino *et al.* (2022) and GenomeScope v.2.0 (Vurture *et al.*, 2017). The *k*-mer distribution was generated by KMC v.3 (Kokot *et al.*, 2017) with default settings (*k*-mer = 21) and the ‘*-cx1000000*’ option to account for the high-frequency *k*-mers derived from repeat content in the genome. The updated genome size estimation was 421 Mb (Supplementary Data Fig. S2), higher than the previously reported size of 325 Mb by Garassino *et al.* (2022), which was found using GenomeScope v.1.0.

The *H. incana* draft genome v.1.0 (Garassino *et al.*, 2022) was used as a starting point for assembly (Supplementary Data Fig. S2). ONT trimmed reads of a minimum of 10 kb were used for genome scaffolding using ntLink v.1.3.9 (Coombe *et al.*, 2023) with the options ‘*gap-fill*, *k=32, w=500*’. The ONT-derived assembly was polished for two rounds by RACON v.1.4.3 (Vaser *et al.*, 2017) using ONT data from this study and Illumina data from Garassino *et al.* (2022). The resulting ONT genome assembly is labelled v.1.5. Assembly v.1.5 and Hi-C reads were used as input data for chromosome-level scaffolding using Juicer v.1.6 (Durand *et al.*, 2016*b*) with default settings and ‘*-s none*’ for DNAse-treated data. The output file ‘*merged_nodups*’ was subjected to the 3D-DNA pipeline v.201008 (https://github.com/theaidenlab/3d-dna) with options ‘*-i 100000 --sort-output*’ to obtain the final genome assembly (v.2.0). In brief, during this step, Juicer first produced contact maps (i.e. genome 3D interactions) based on the Hi-C data, then 3D-DNA software used them to scaffold, detect and correct genome mis-assemblies. These mis-assemblies could be split, reordered and rejoined to maximize the consistency with the observed Hi-C interactions in the contact maps. This resulted in an increase in scaffold number from 246 (v.1.5) to 358 (v.2.0), which generally improved the assembly statistics, including N50 (the length of the shortest scaffold at which 50% of the genome assembly is contained in scaffolds of that length or longer) and L90 (the minimum number of scaffolds represents 90% assembly) of v.2.0 (see Table 1). The Hi-C contact map was visualized and reviewed by Juicebox v.2.20.00 (Durand *et al.*, 2016*a*). Genome quality and completeness were analysed by QUAST v.5.2.0 (Gurevich *et al.*, 2013) and BUSCO (Benchmarking Universal Single-Copy Orthologs) v.5.4.7 (Simao *et al.*, 2015) based on the plant-specific Embryophyta odb10 dataset, which includes 1614 single-copy orthologues. Mapping back rates were obtained by mapping a total 96 million WGS read data from Garassino *et al.* (2022) using Bowtie2 v.2.5.1 (Langmead and Salzberg, 2012) with parameters ‘*--very-sensitive --no-unal -k 20*’. These reads were derived from a total 102 million reads after the removal of contamination reads that mapped onto the *H. incana* chloroplast and mitochondrial genomes (Garassino *et al.*, 2022) by bbduk.sh (http://sourceforge.net/projects/bbmap) with options ‘*k=31 hdist=1*’. Genome circular plots were drawn using Circos v.0.69-9 (Krzywinski *et al.*, 2009).

**Table 1.** Summary statistics of the genome assembly and annotation of *Hirschfeldia incana* and relatives

Genome features	*Brassica rapa* v.4.0 (Chiifu)	*Brassica oleracea* v.2.0 (JZS)	*Brassica nigra* v.2.0 (NI100)	*Hirschfeldia incana* v.1.0 (NIJ)	*Hirschfeldia incana* v.1.5 (NIJ)	*Hirschfeldia iincana* v.2.0 (NIJ)
Chromosome number (*n* = *x*)	10	9	8	7	7	7
Assembled genome size (Mb)	424.59	561.16	506.00	398.50	408.86	408.93
GC content (assembly, %)	37.59	36.75	38.21	36.18	36.20	36.20
Number of scaffolds	10	649	58	384	246	358
Number of pseudomolecules	10	9	8	N/A	N/A	7
Scaffold N50 length (Mb)	43.05	57.88	60.82	5.11	13.76	52.38
Scaffold N90 length (Mb)	30.43	47.84	55.08	0.83	1.99	48.08
Longest scaffold (Mb)	73.37	74.51	70.85	15.00	30.16	63.71
N’s per 100 kb	0.05	26.05	2.47	13.45	1.70	18.08
BUSCO assembly (%)	99.5	99.5	99.2	99.3	99.5	99.2
Number of genes	47 531	59 064	59 852	32 313	54 459	54 457
GC content (main transcript, %)	46.24	46.13	45.92	46.53	46.19	46.19
N50 length (main transcript, bp)	1473	1428	1449	1515	1377	1377
Mean length (main transcript, bp)	1147	1155	1026	1247	1035	1035
BUSCO (main transcripts, %)	97.2	98.7	98.2	96.2	97.8	97.7

### Transcriptome sequencing, transcript assembly and other data acquisition

To aid genome annotation, we used the ONT sequencing technology to generate long-read transcriptome data for six samples of *H. incana* leaves collected from two conditions, low-light (200 µmol m^−2^ s^−1^) and high-light (1800 µmol m^−2^ s^−1^), 6 weeks after planting. Leaf samples were collected from the third to fifth compound leaves, counting from the top of each plant (i.e. functional leaves), snap-frozen in liquid N_2_ and stored at −80 °C until further processing.

RNA isolation was performed following the TRIzol™ Reagent RNA isolation protocol (Thermo Fisher Scientific, Waltham, MA, USA) combined with the RQ1 RNase-Free DNase Protocol (Promega, Madison, WA, USA) using ~100 mg of ground tissue. The Nanopore sequencing library preparation was conducted following the PCR-cDNA Barcoding Kit (SQK-PCB111.24) protocol. Sequencing was performed on the MinION Mk1B platform using flow cells (FLO-MIN106D) and was base-called with Dorado simplex v.0.3.4 (https://github.com/nanoporetech/dorado). Chopper v.0.8.0 (https://github.com/wdecoster/chopper) was used for trimming the raw reads, which were then used as direct evidence in our genome annotation pipeline. For assessment of data quality, see Supplementary Data Fig. S1.

We also obtained the *H. incana* leaf transcriptome data from Mabry *et al.* (2020) and Garassino *et al.* (2022) and whole canopy data from Garassino *et al.* (2024). These Illumina short-reads and their assembled transcripts were used during genome annotation and quality checking. Transcriptomes were assembled using the Trinity pipeline v.2.15.0 (Haas *et al.*, 2013) with default settings. Additionally, the previously assembled transcripts for *H. incana* samples representing above- and below-ground tissues obtained from Hasnaoui *et al.* (2022) were also used in genome annotation. It is important to note here that the data from Garassino *et al.* (2022) and (2024) were from the same *H. incana* NIJ accession, whereas data from Mabry *et al.* (2020) and Hasnaoui *et al.* (2022) were derived from different accessions.

### *Repeat and gene annotation of the* H. incana *genome*

Repeats and transposable elements in the genome were masked with RepeatModeler v.2.0.3/RepeatMasker v.4.1.2 and RepeatProteinMask (Tarailo-Graovac and Chen, 2009). Firstly, the *ab initio* prediction program RepeatModeler was employed to build a *de novo* repeat library based on the *H. incana* genome. Then, using a custom library that consisted of *de novo* identified repeats, Dfam v.3.3 and RepBaseRepeatMaskerEdition-20181026 as the database, RepeatMasker was run to find and classify repetitive elements in the genome. The centromere location of each chromosome was identified following the approach used for radish genomes (Jeong *et al.*, 2016; Cho *et al.*, 2022). Briefly, the centromeric tandem repeats (CENTs) from *R. sativus* and *Brassica* genomes were blasted against the *H. incana* genome with an E-value cut-off of 1 × 10^−10^. The blast hit results were analysed to identify the likely centromeric regions of the *H. incana* chromosomes.

For *de novo* gene prediction, the PASA pipeline v.2.4.1 (Haas *et al.*, 2008) was used to train a model using the available RNA-sequencing (RNA-seq) data as direct evidence, and the resulting PASA models were then used to train AUGUSTUS v.3.1.0 (Stanke and Morgenstern, 2005). Other *de novo* gene predictions were performed using SNAP v.20131129 (Korf, 2004), GeneMark v.4.72 (Bruna *et al.*, 2020) and GlimmerHMM v.3.0.4 (Majoros *et al.*, 2004). Protein evidence used during gene prediction was collected from the UniProtKb/SwissProt curated protein database release 2024_02 (UniProt Consortium, 2015), the Viridiplantae dataset from OrthoDB v.11 (Kuznetsov *et al.*, 2023) and proteins from related species, including *A. thaliana*, *B. rapa*, *B. oleracea*, *B. nigra* and *H. incana* v.1.0. We combined the predicted gene models from different programs, transcript evidence and protein evidence to produce consensus gene sets by using EvidenceModeler v.2.1.0 (Haas *et al.*, 2008) standalone and within the Funannotate pipeline v.1.8.16 (https://github.com/nextgenusfs/funannotate). This combination step was performed by giving different weights to different predictions, as follows: ‘*--weights augustus:2 pasa:10 snap:1 transcripts:6 proteins:3 GeneMark:1 GlimmerHMM:1*’, to reduce spurious gene prediction by *de novo* prediction programs. The BRAKER v.3.0.7 (Hoff *et al.*, 2019) and HELIXER v.0.3.2 (Holst *et al.*, 2023) annotation pipelines were also tested and compared with the results from EvidenceModeler and Funannotate pipelines to choose the best annotation set. The final annotation was updated by PASA to add data for untranslated regions and to fix gene models that were not in agreement with the RNA-seq data. Initially, the annotation was done for the ONT-derived genome assembly v.1.5, then lifted over to the final Hi-C genome assembly (v.2.0) using LIFTOFF v.1.6.3 (Shumate and Salzberg, 2021). The BUSCO assessment based on 1614 Embryophyta single-copy orthologues and OMArk v.0.3.0 based on 17 999 conserved orthologues of the Brassicaceae family (Nevers *et al.*, 2024) were used to analyse and compare the annotations.

### Gene functional annotation

The *H. incana* predicted proteins were blasted against the Swiss-Prot release 2022_04 (O’Donovan *et al.*, 2002) and TrEMBL release 2022_01 (O’Donovan *et al.*, 2002) using Diamond BLASTP v.2.0.14 (Buchfink *et al.*, 2021) with the following settings ‘*-e 1e-5 -k 1*’. To predict protein function [both protein domains and associated gene ontology (GO) terms], we used InterProScan-5.66-98.0 (Zdobnov and Apweiler, 2001) to blast the *H. incana* proteins against several databases with the options‘*-goterms*’. We used all 17 databases provided with InterProScan to maximize the annotation. Kyoto Encyclopedia of Genes and Genomes (KEGG) mapping was done using BlastKOALA v.2.2 (Kanehisa *et al.*, 2016) with ‘*plants*’ as taxonomy group and searched against the ‘*family_eukaryotes*’ KEGG gene databases.

### Orthogroup classification

To infer the orthology of *H. incana* and other Brassicaceae genomes, primary (longest variant) protein sequences were used for orthogroup clustering by OrthoFinder v.2.5.5 (Emms and Kelly, 2019) with default settings and the ‘-*M msa*’ option to infer maximum likelihood (ML) gene trees from multiple sequence alignment. Additionally, OrthoFinder was used for identification of single-copy orthologues across genomes and reconstruction of a species tree based on the identified single-copy orthologues. This species tree was used to compare with those generated from IQ-TREE v.2.2.0 (Minh *et al.*, 2020) and ASTRAL v.5.7.1 (Zhang *et al.*, 2018) to infer the relationship between *H. incana* and other species within the Brassiceae used in this study (for details, see the ‘Nuclear phylogenetic analyses’ section).

### Genome synteny and duplication analyses

Macro- and micro-synteny of the genomes of *H. incana* and other Brassicaceae species were analysed by SynMap (Lyons *et al.*, 2008), SynFind (Tang *et al.*, 2015) on the CoGe v.7 (Castillo *et al.*, 2018) and MCscan v.0.8 (Tang *et al.*, 2008) python version (https://github.com/tanghaibao/jcvi/wiki/MCscan-(Python-version)). Additionally, MCScanX (accessed December 2023) (Wang *et al.*, 2012) and DupGen_finder (accessed December 2023) (Qiao *et al.*, 2019) were used for various analyses. Modes of duplicated gene copies were analysed by DupGen_finder with default parameters, using the *A. thaliana* genome as reference. For each Brassiceae genome, gene duplications were classified into WGD/WGT, tandem, proximal, transposed, and dispersed duplicates.

### Estimation of Ks ratios of WGD/WGT duplicated gene pairs

The *Ks* (the ratio of number of substitutions per synonymous site) values were computed for WGT/WGD gene pairs identified by DupGen_finder using *KaKs*_Calculator v.2.0 (Wang *et al.*, 2010) following the pipeline in the study by Qiao *et al.* (2019). This used MAFFT v.7.480 (Katoh *et al.*, 2002) and PAL2NAL v.14 (Suyama *et al.*, 2006) and the γ-MYN method (Wang *et al.*, 2009*a*). To identify the *Ks* peaks corresponding to the recent WGD/WGT events in the *H. incana* genome, we fitted the *Ks* distribution using a Gaussian mixture model, as described in Qiao *et al.* (2019). To infer species divergence, *Ks* values between syntenic gene pairs were also calculated by CodeML (Yang, 2007) in SynMap running on the CoGe v.7 (https://genomevolution.org/coge/).

### *Ancestral genomic blocks and karyotype evolution of the* H. incana *genome*

The ancestral tPCK genomic blocks (Schranz *et al.*, 2006; Lysak *et al.*, 2016) were used to analyse the *H. incana* genome structure. We used the updated genomic blocks for the *Brassica* genomes in the study by He *et al.* (2021) to determine the intervals and boundaries of the 26 ancestral genomic blocks in the *H. incana* genomes. The triplicated *H. incana* genomic blocks were then classified into three sub-genomes (LF, MF_1_ and MF_2_) based on the gene retention rate compared with the ancestral blocks.

Briefly, we aligned the *H. incana* genome to the 26 tPCK ancestral genomic blocks (as target/reference) using SynMap. This analysis was run together with the program FractBias (Joyce *et al.*, 2017) on the CoGe v.7 using a window size of 100 genes. The fractionation bias rate was calculated for syntenic genes in the target genome. Syntenic depth was set to 1:3 based on the ploidy level between the ancestral genomes and *H. incana*. Additionally, to elucidate genome rearrangement among *H. incana*, *R. sativus*, *S. arvensis* and three *Brassica* A/C/B genomes, we used the IAGS pipeline (accessed February 2024) (Gao *et al.*, 2022) to reconstruct their common ancestral genome using the orthologous results from OrthoFinder and the non-overlapping syntenic blocks detected by Drimm-Synteny (accessed February 2024) (Pham and Pevzner, 2010).

### Nuclear phylogenetic analyses

To reconstruct the species tree, single-copy orthologues were identified by OrthoFinder v.2.5.5 across selected genomes as described earlier. This used MAFFT v.7.480 (Katoh *et al.*, 2002) for sequence alignment and FastTree v.2 (Price *et al.*, 2009) for the phylogenetic tree inference. For IQ-TREE analysis, coding or protein sequences were aligned by MAFFT with the option ‘*G-INS-i*’, then poorly aligned regions were trimmed by trimAL v.1.4.rev22 (Capella-Gutierrez *et al.*, 2009) with the option ‘*-automated1*’. The alignment files then were subjected to IQ-TREE v.2.2.0 (Trifinopoulos *et al.*, 2016) with default settings (1000 bootstrap iterations).

For phylogenetic incongruency analyses, coding sequences from the 5675 ‘strict single-copy genes’ identified among six species (*A. thaliana*, *B. rapa*, *B. oleracea*, *B. nigra*, *R. sativus* and *H. incana*) using OrthoFinder were aligned using MACSE v.2.06 (Ranwez *et al.*, 2018). Gene trees were reconstructed using RAxML-NG v.1.1.0 (Kozlov *et al.*, 2019) with substitution model GTR+G and 1000 bootstrap replicates, while setting *A. thaliana* as the outgroup. A species tree was reconstructed with ASTRAL v.5.7.8 (Zhang *et al.*, 2018) based on all gene trees. The tree topology was then used as input for DensiTree v.3.0.3 (Bouckaert, 2010) and PhyParts v.0.0.1 (Smith *et al.*, 2015) to assess conflict among the gene trees. For each bipartition, the software assesses the number of gene trees that support the main topology, the most common alternative topology, all other topologies, and the number of gene trees that are not informative for the respective bipartition. Here, we also used three support levels (no threshold, bootstrap support of <50 %, and bootstrap support of <85 %) to count as uninformative. PhyPartsPieCharts (https://github.com/mossmatters/phyloscripts/tree/master/phypartspiecharts, accessed February 2024) was used for visualization.

To reconstruct a sub-genome tree, genes retained in all three sub-genomes (triads) across all species were obtained from SynMap analysis using the ancestral block as reference against the target genomes. We focused our analysis on 90 genes from shared ancestral block F. Codon-aware alignments were created using MACSE v.2.06, and gene trees were reconstructed using RAxML-NG v.1.1.0 as described above. We then reconstructed two different types of trees. First, a species tree was reconstructed using ASTRAL-pro v.1.15.1.3 (Zhang *et al.*, 2020), which allows for multi-copy genes; here, the three gene copies in each Brassiceae species were considered paralogues. Second, a sub-genome tree was reconstructed using ASTRAL v.5.7.8, in which the three copies in each species were assigned to their respective sub-genomes (LF, MF_1_ and MF_2_). The final consensus trees were visualized by FigTree v.1.4.3 (http://evomics.org/resources/software/molecular-evolution-software/figtree/).

### Endoreduplication analysis by flow cytometry

Endoreduplication (endopolyploidy) analysis was performed using leaf samples from three species [*B. rapa* (R-o-18 accession), *B. nigra* (DG1 accession) and *H. incana* (NIJ accession)] by flow cytometry (Plant Cytometry, Didam, The Netherlands). Leaf samples were collected from plants grown under low-light (200 µmol m^−2^ s^−1^) and high-light (1800 µmol m^−2^ s^−1^) at 30 days after sowing. Three leaf developmental stages (very young, young and mature) were used.

### Gene expression analysis

For analysis of gene expression in leaf tissues, we used whole canopy transcriptome data reported by Garassino *et al.* (2024) from two contrasting light conditions, low-light (200 µmol m^−2^ s^−1^) and high-light (1800 µmol m^−2^ s^−1^). RNA-seq read quality before and after trimming was assessed by FastQC v.0.11.9 (http://www.bioinformatics.babraham.ac.uk/projects/fastqc). Adapter sequences and low-quality reads were trimmed using Trimmomatic v.0.39 (Bolger *et al.*, 2014) and the following parameters: ‘ILLUMINACLIP: 2:20:10 SLIDINGWINDOW:4:15 LEADING:5 TRAILING:5 MINLEN:50’. To estimate transcript abundance, cleaned reads were mapped onto *H. incana* gene models using Bowtie2 v.2.4.5 (Langmead and Salzberg, 2012) with default settings. The mapping BAM files were sorted by SAMTOOLS-1.19.2 (Li *et al.*, 2009) and subjected to RSEM v.1.3.3 (Li and Dewey, 2011) for quantification of transcript abundance, normalized as transcripts per million transcripts (TPM). Differentially expressed genes were identified using the DESeq2 package (Love *et al.*, 2014), with a false-discovery rate (FDR)-corrected *P*-value ≤ 0.05 and |fold-change| ≥ 2. Additionally, we used Mercator v.4.6 (Lohse *et al.*, 2014) for functional annotation and categorization of the identified differentially expressed genes.

### Other quantification and statistical analyses

Venn diagrams were generated using the online tools (http://bioinformatics.psb.ugent.be/webtools/Venn) and InteractiVenn (Heberle *et al.*, 2015). GO term and KEGG pathway enrichment of gene sets were performed using the DAVID bioinformatics resources v.2023q4 (Huang *et al.*, 2009) and ShinyGO v.0.80 (Ge *et al.*, 2020). All analyses in the Linux environment were performed on local servers running Ubuntu 16.04.6 LTS hosted by the Biosystematics Group at Wageningen University, The Netherlands. All statistical analyses, unless otherwise stated, were performed in Microsoft Excel v.18.2311.1071.0 and R v.4.0.2 with RStudio v.2022.07.2-576 (https://www.rstudio.com).

## RESULTS AND DISCUSSION

### *A chromosome-level assembly and re-annotation of the* H. incana *genome*

The scaffold-level genome assembly v.1.0 of the NIJ accession of C_3_ species *H. incana* was previously reconstructed based on PacBio SMRT long-read, 10× Genomics linked-read, and Illumina short-read data (Garassino *et al.*, 2022). Here, we first improved the draft genome assembly v.1.0 through two rounds of scaffolding using ONT and Hi-C sequencing, respectively. We generated a total of ~20 Gb of long-read ONT data (N50 = 26 kb, 48× genome coverage) and 124 million Hi-C Illumina reads (150 bp, 44× genome coverage) (Supplementary Data Table S1; Fig. S1). The overlapping ONT data that span through v.1.0 scaffolds were used to link them into larger sequences to obtain an ONT-derived assembly (termed v.1.5). We subsequently used v.1.5 as input for a second round of scaffolding based on the Hi-C data to produce the final chromosome-level genome assembly (termed v.2.0).

Compared with the previous assembly, v.1.0 (size 399 Mb, scaffold N50 of 5 Mb), both v.1.5 and v.2.0 have a slightly larger assembly size of 409 Mb, and significantly improved N50 lengths, of 14 and 52 Mb, respectively (Table 1). Overall, the size of the three assemblies is close to our re-estimated genome size of 421 Mb using *k*-mer analysis (Supplementary Data Fig. S2 and Methods) and smaller than the flow cytometry estimate of 487 Mb (Garassino *et al.*, 2022). Our v.2.0 assembly N50 length (52 Mb) and chromosome size are comparable to other chromosome-level assemblies of related Brassiceae species, including *B. rapa* v.4.0 and v.4.1 (Zhang *et al.*, 2023), *B. oleracea* JZS v.2.0 (Cai *et al.*, 2020) and *B. nigra* N100 v2.0 (Perumal *et al.*, 2020), with N50 lengths ranging from 43 to 61 Mb (Supplementary Data Table S2) and individual chromosome sizes of 30–75 Mb (Supplementary Data Fig. S3).

**Fig. 1.**
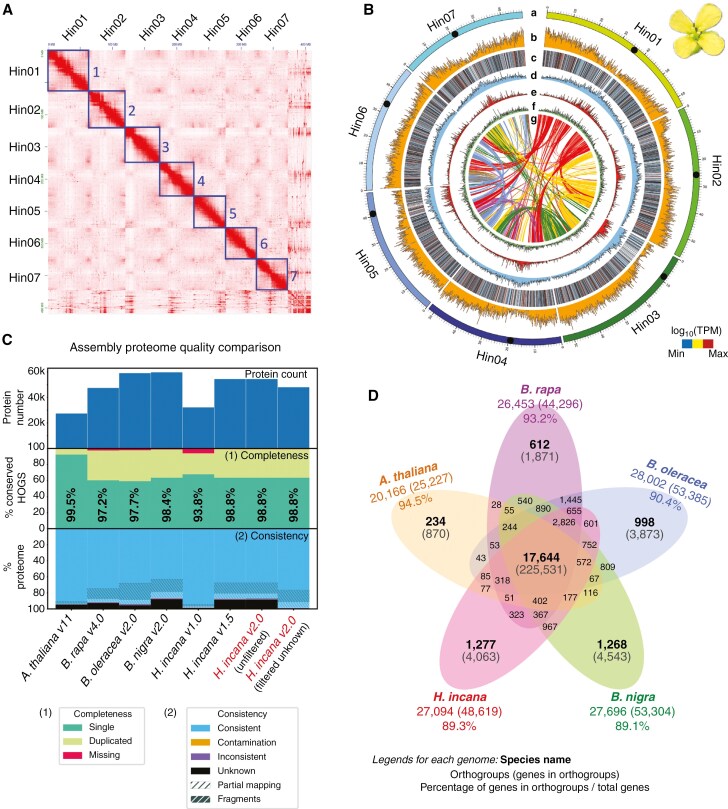
The assembly of *Hirschfeldia incana* NIJ genome v.2.0, intra-genomic synteny, quality assessment and gene orthogroup clustering. (A) Chromosome-level Hi-C contact maps of the *H. incana* genome, highlighting seven blocks that correspond to its seven chromosomes. (B) Circos plot showing seven chromosomes of the *H. incana* genome (track a), gene density (track b), gene expression of the predicted gene models (track c), distribution of all repetitive elements (track d), distribution of LTR elements (track e), DNA transposon elements (track f) and intra-genomic synteny (*minspan = 4 genes*) (track g). Distributions were estimated for each window of 100 kb. Gene expression was calculated using the transcriptome data from Garassino *et al.* (2024) as log_10_[average transcripts per million transcripts (TPM)] over all ten samples from two conditions, low-light and high-light. Ribbon links in the inner track of the Circos plot represent intra-genomic syntenic regions among chromosomes. For a dotplot, see Supplementary Data Fig. S9. Centromere regions are indicated by black dots on each chromosome. Length is in megabases. (C) Comparisons of proteomes among selected Brassicaceae genomes by OMArk tool, including proteome count, completeness and annotation consistency against a total of 17 999 conserved orthologues of the Brassicaceae family in the OMA database. Percentage of completeness for each genome is the total percentage of single and duplicated completeness of the hierarchical orthologous groups (HOGs). For *H. incana* v.2.0, two proteome datasets, unfiltered and filtered, are shown. The filtered dataset was obtained from the initial proteome after the removal of unknown proteins that were not found in the OMA database. (D) Venn diagram showing shared and unique orthogroups in *Arabidopsis thaliana*, *Brassica rapa*, *Brassica oleracea*, *Brassica nigra* and *H. incana*. Numbers in parentheses denote genes included in the orthogroups (as explained in the figure). Percentages were calculated based on the total genes annotated in each selected genome. For panels C and D, the genomes of *A. thaliana* Col-0, *B. rapa* Chiifu, *B. oleracea* JZS, *B. nigra* NI100 and *H. incana* NIJ were used (for more details, see the Materials and Methods).

The final genome assembly, v.2.0, has 358 scaffolds, with the majority (91.2 % assembly length and 96 % predicted genes) anchored onto seven super scaffolds (Fig. 1A, B; Table 1) which correspond to the seven (*x* = 7) reported chromosomes for *H. incana* (Garassino *et al.*, 2022). All seven chromosomes contain centromere-specific repeat sequences (CENTs; Jeong *et al.*, 2016; Cho *et al.*, 2022), detected within the repeat-rich regions (Fig. 1B). By mapping back the 96 million WGS Illumina reads generated by Garassino *et al.* (2022), it was found that all three assemblies have comparable mapping rates of ~96 % (Supplementary Data Table S3). The BUSCO completeness score (Simao *et al.*, 2015) of all three assemblies ranged from 99.2 to 99.5 % (Supplementary Data Table S4). This indicates that we successfully incorporated v.1.0 sequences into our final chromosome-level assembly, v.2.0, and all three assemblies represent the gene content of the *H. incana* genome well.

As expected, the repeat content of the *H. incana* genome assembly v.2.0 is very similar to that of v.1.0, with 50.3 % of the sequences being masked as repetitive elements, present in two major classes, the long terminal repeat retrotransposons (LTR-RT) and DNA transposons (Fig. 1B; Supplementary Data Table S5). By integrating various gene prediction approaches, we annotated a total of 54 457 protein-coding gene models (59 417 total transcripts) and 1262 transfer RNAs (Supplementary Data Figs S4 and S5) with a 97.7 % BUSCO completeness score (Supplementary Data Table S6). Overall, the total number of gene models in genome annotation v.2.0 is higher than that reported for v.1.0 (32 313) by Garassino *et al.* (2022) but similar to those of other Brassiceae genomes (47 000–60 000; see Table 1). Our annotation, therefore, better reflects the *Br-α* WGT history of the *H. incana* genome, particularly when compared with the gene models predicted for the *A. thaliana* genome (~27 000), which did not experience the WGT event. Nevertheless, the annotation v.2.0 is highly syntenic with v.1.0, as indicated by the syntenic path assembly in their dotplot (Supplementary Data Fig. S6). Most of the gene models (99.9 %) matched with sequences in at least one of the public protein databases or assembled transcripts (Supplementary Data Table S7), including 71.9 % matching with Swiss-Prot (O’Donovan *et al.*, 2002), 91.2 % with TrEMBL (O’Donovan *et al.*, 2002), 74.2 % with InterPro (Zdobnov and Apweiler, 2001), 62% with GO (Ashburner *et al.*, 2000), 55.0 % with KEGG (Kanehisa and Goto, 2000), 88.4 % with OMA database (Nevers *et al.*, 2024), and 87.9 % with assembled transcripts from *H. incana*.

To assess our gene annotation further, we used the OMArk tool (Nevers *et al.*, 2024) to compare our proteome completeness and consistency with that of four closely related species using 17 999 conserved hierarchical orthologous groups of the Brassicaceae family. The completeness score of the *H. incana* proteome v.2.0 was 98.8 % (compared with 93.8 % for v.1.0) and comparable to that of other Brassicaceae species proteomes (Fig. 1C; Supplementary Data Fig. S7; Table S8). The percentage of our proteome that matched the conserved Brassicaceae hierarchical orthologous groups in the OMA database is slightly higher than *B. nigra* (88.4 vs. 87.7 %), but lower than *B. rapa*, *B. oleracea* and *A. thaliana* (92.8, 95.7 and 94.7 %, respectively). Additional orthologue clustering by OrthoFinder (Emms and Kelly, 2019) of our proteome and the proteomes of the aforementioned Brassicaceae genomes resulted in 48 619 *H. incana* genes (89.3 % of total genes) being classified into 27 094 orthogroups, of which 17 644 were commonly shared with four other Brassicaceae proteomes (Fig. 1D; Supplementary Data Table S9). Collectively, these assessments indicate that our improved genome assembly and annotation of *H. incana* v.2.0 is of good quality and could be used together with that of its Brassiceae relatives for synteny-based and trait evolution studies of high photosynthetic rates in the Brassiceae tribe.

### *The* H. incana *genome exhibits the typical triplicated structure of the Brassiceae tribe*

**Fig. 2.**
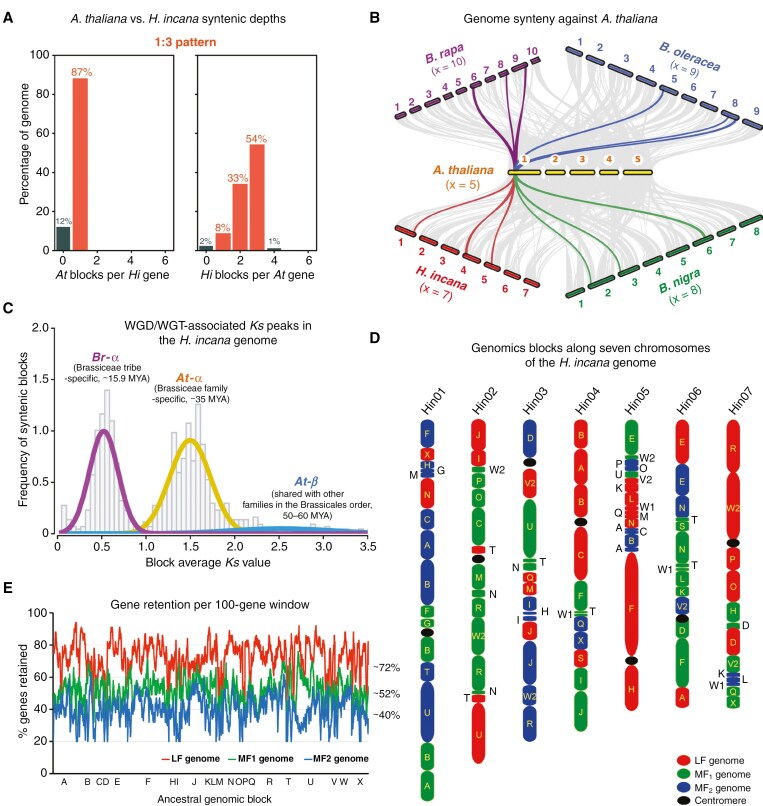
Genomic architecture of the *Hirschfeldia incana* NIJ genome. (A) Ratio of syntenic depth between *Arabidopsis thaliana* and *H. incana*. Syntenic blocks of *A. thaliana* per *H. incana* gene (left) and syntenic blocks of *H. incana* per *A. thaliana* gene (right) are shown which suggest a clear 1:3 pattern between the two genomes. (B) Genome macro-synteny between *A. thaliana* and four Brassiceae genomes (*Brassica rapa*, *Brassica oleracea*, *Brassica nigra* and *H. incana*) that underwent the *Br-α* whole-genome triplication (WGT) event, showing a similar 1:3 syntenic pattern. A syntenic block located on *A. thaliana* chromosome 1 was chosen to illustrate the triplicated pattern in the Brassiceae genomes. The genomes of *A. thaliana* Col-0, *B. rapa* Chiifu, *B. oleracea* JZS, *B. nigra* NI100 and *H. incana* NIJ were used. (C) WGD/WGT events identified in the *H. incana* genome by fitting the *Ks* distributions for WGD/WGT-derived gene pairs using a Gaussian mixture model. *Ks* peaks correspond to the *At-β* (shared with other families within the Brassicales), *At-α* (Brassicaceae-specific) and *Br-α* (Brassiceae-specific) events. Only *Ks* ≤ 3.5 were included in this analysis. Date estimates were derived from Edger *et al.* (2018), Kagale *et al.* (2014), Liu *et al.* (2014) and Jiao *et al.* (2011). (D) Ancestral genomic blocks along the seven *H. incana* chromosomes. The updated tPCK ancestral genome from He *et al.* (2021) was mapped onto the *H. incana* genome to identify intervals of the triplicated conserved genomic blocks, then these triplicated blocks were classified into three sub-genomes (LF, MF_1_ and MF_2_) based on their gene retention pattern analysed by FractBias (Joyce *et al.*, 2017). Sub-genomes were coloured red, green and blue, respectively. Centromeres are represented by black ovals. (E) Gene fractionation bias in the three *H. incana* sub-genomes. The ancestral tPCK genomic blocks were used as reference. Gene retention (as a percentage) was calculated in sliding windows of 100 genes across the tPCK genomic blocks based on all identified syntenic genes.

The meso-hexaploidy *Br-α* WGT event was previously reported based on the genomes of several Brassiceae species, including those from the genera *Brassica* (Wang *et al.*, 2011; Liu *et al.*, 2014; Perumal *et al.*, 2020), *Raphanus* (Jeong *et al.*, 2016; Xu *et al.*, 2023) and *Sinapis* (Yang *et al.*, 2023). As a member of the Brassiceae tribe, it is expected that the *H. incana* genome also underwent the *Br-α* WGT event. Here, using the updated chromosome-scale assembly and annotation and the total syntenic gene pairs between *A. thaliana* and *H. incana*, we find a clear 1:3 syntenic pattern between the two respective species. More specifically, 87 % of the *H. incana* genes have one syntenic block in the *A. thaliana* genome, while 8, 33 and 54 % of the *A. thaliana* genes have one, two and three syntenic blocks in the *H. incana* genome, respectively (Fig. 2A; Supplementary Data Fig. S8). Those *H. incana* genes within the three detected syntenic blocks are likely to be located within the well-retained genomic regions, whereas *H. incana* genes found in one or two syntenic blocks are likely to be those within more fractionated regions. The syntenic relationship between *H. incana* and *A. thaliana* genomes resembles the relatedness between each of the three *Brassica* genomes (*B. rapa*, *B. oleracea* and *B. nigra*) and *A. thaliana*, as illustrated in Fig. 2B. Additionally, a clear triplicated intra-genomic syntenic pattern could be observed within the *H. incana* genome (Supplementary Data Fig. S9), while a three-to-three (or one-to-one, if only true orthologues were considered) syntenic relationship between *H. incana* and *B. rapa* was observed when comparing the two genomes (Supplementa Data Fig. S10). Our results support the notion that the *H. incana* genome, like other Brassiceae genomes, also experienced the Brassiceae tribe-specific hexaploidy *Br-α* WGT event.

We elucidated the WGD/WGT history of *H. incana* further by fitting the distribution of *Ks* values from its WGD/WGT-derived gene pairs (Supplementary Data Fig. S11) using a Gaussian mixture model (Qiao *et al.*, 2019). Here, we revealed three major *Ks* peaks corresponding to the three recent WGD/WGT events in the genome (Fig. 2C). The youngest peak (in purple) represents the more recent *Br-α* WGT event that was shared among the Brassiceae species (Brassiceae tribe specific, ~15.9 Mya), while the two more ancient peaks (in yellow and blue) respectively represent the more ancient *At-α* WGD event (Brassicaceae family specific, ~35 Mya, i.e. late Eocene to early Oligocene epochs) and *At-β* WGD event (shared among Brassicaceae and other families in the Brassicales order, 50–60 Mya, i.e. the mid-Palaeocene to early Eocene epochs) (Jiao *et al.*, 2011; Kagale *et al.*, 2014; Liu *et al.*, 2014; Edger *et al.*, 2018). The fitted peaks are consistent with those identified in the *Brassica* genomes previously reported using the same method (Qiao *et al.*, 2019; Yim *et al.*, 2022; Hoang *et al.*, 2023).

The Brassiceae genomes are known to be derived from the ancestral tPCK karyotype (Schranz *et al.*, 2006; Lysak *et al.*, 2016). We thus used the updated ancestral genomic blocks of the *Brassica* genomes (He *et al.*, 2021) to determine the intervals and boundaries of the 26 ancestral genomic blocks in the *H. incana* genome (Fig. 2D; Supplementary Data Table S10; Figs S12 and S13). We were able to identify most of the triplicated blocks (except small blocks G, S and V1) within the *H. incana* genome that are syntenic to the ancestral genomic blocks. Based on the gene retention rate, these triplicated genomic blocks were then classified into three sub-genomes, the LF, MF_1_ and MF_2_ (Fig. 2D). The average retention rates of these sub-genomes compared with the ancestral tPCK genome were 72, 52 and 40 % for LF, MF_1_ and MF_2_, respectively (Fig. 2E). This sub-genome biased fractionation, as a result of the two-step polyploidization process, has been reported in other Brassiceae genomes (Cheng *et al.*, 2012; Liu *et al.*, 2014; Perumal *et al.*, 2020; Cho *et al.*, 2022; Yang *et al.*, 2023), underscoring the notion that the *H. incana* genome was also derived from the common tPCK ancestral genome of the Brassiceae tribe. By reconstructing an intermediate common ancestral genome of the six Brassiceae species, we found that, compared with the genomes of other Brassiceae species, the *H. incana* genome showed a similar level of rearrangement to that of *B. nigra* and *S. arvensis* (36–41 fissions and 38–44 fusions), but higher than that of *B. rapa*, *B. oleracea* and *R. sativus* (29–32 fissions and 31–33 fusions) (Supplementary Data Fig. S14).

Taken altogether, the updated genome assembly of *H. incana* allowed us to elucidate its genome structure, WGD/WGT history and genome evolution at the sub-genome level. The *H. incana* genome was derived from the recent Brassiceae-specific *Br-α* WGT event that resulted in three distinct sub-genomes that display differential gene retention rates. These sub-genomes were shown to have originated from the ancestral tPCK karyotype of the Brassiceae, similar to that of the other genomes from the *Brassica*, *Raphanus* and *Sinapis* genera.

### *Phylogenomic analysis reveals* H. incana *(H) and* R. sativus *(R) are sister to the* Brassica *B genome type clade*

Several chromosome-level genome assemblies of Brassiceae species have been released, including those outside of the *Brassica* ‘triangle of U’, such as *R. sativus* (Cho *et al.*, 2022; Xu *et al.*, 2023) and *S. arvensis* (Yang *et al.*, 2023). Large-scale comparative genomics and phylogenetic analyses have provided insights into relationships among these species. However, owing to the rampant hybridization among Brassiceae species that hinders phylogenetic inference, a consensus phylogenetic tree for this tribe is not yet resolved. Although the relationships between the *Brassica* A/C and B genome types appear to be more consistent in published nuclear phylogenetic trees, this is not the case for *R. sativus* (Huang *et al.*, 2016; Cho *et al.*, 2022; Yang *et al.*, 2023) and *H. incana* (Huang *et al.*, 2016; Garassino *et al.*, 2022; Guerreiro *et al.*, 2023), because these two species were placed closer to either *Brassica* A/C or B genome type species. Interestingly, Jeong *et al.* (2016) and Cho *et al.* (2022) revealed that the genome structure of *R. sativus* displayed intermediate characteristics between A/C and B genome types.

**Fig. 3.**
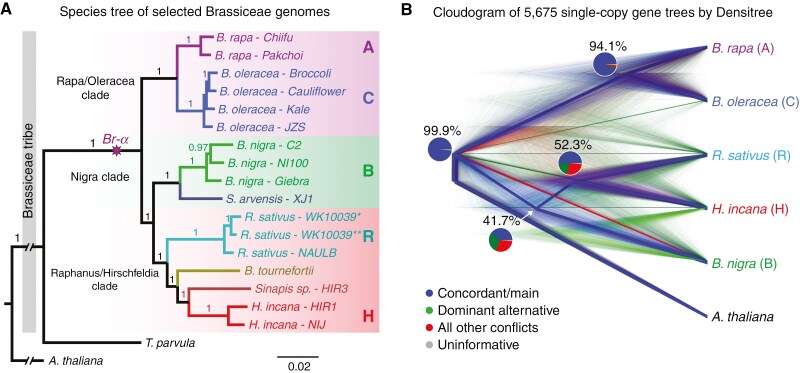
Phylogenetic relationships of *Hirschfeldia incana* and other Brassiceae species. (A) Species tree of ten selected Brassiceae species, including several genome accessions for each species. The tree was reconstructed based on 1504 single-copy genes identified across the selected genomes. Tree was rooted using the *Arabidopsis thaliana* (and *Thellungiella parvula*) genomes as outgroup. Supporting values at each node are bootstrap scores. Branch length represents the number of substitutions per site. The *Raphanus sativus* WK10039* and ** denotes v.1.0 and v.2.0 assemblies, respectively. (B) Cloudogram of gene trees derived from a total of 5675 single-copy orthologues identified among five selected Brassiceae and the *A. thaliana* genomes showing concordant (main, blue), dominant alternative (green) and other conflict (red) topologies. Pie charts derived from the PhyParts analysis (Smith *et al.*, 2015) show percentage of trees supporting main/alternative/conflict topologies at each node. The tree was rooted using *A. thaliana* as the outgroup. The genomes of *A. thaliana* Col-0, *Brassica rapa* Chiifu, *Brassica oleracea* JZS, *Brassica nigra* NI100, *Sinapis arvensis* XJ1, *R. sativus* NAULB and *H. incana* NIJ were used.

To resolve the discrepancy in the phylogenetic placement of *H. incana* and *R. sativus,* we initially reconstructed a phylogenetic tree using 1504 single-copy nuclear orthologous genes identified across 19 genome accessions from a total of ten species (Fig. 3A). Despite potential problems owing to reciprocal gene loss, single-copy genes have been shown generally to recover species trees even in polyploid clades (Naranjo *et al.*, 2024). These single-copy genes were identified by OrthoFinder, a bioinformatic tool that is conventionally used to identify marker genes for species tree reconstruction in phylogenetic studies, including the aforementioned Brassiceae studies. The ten species included several from the ‘triangle of U’ *Brassica* species (A, C and B genome types), *Raphanus*, *Sinapis* and *Hirschfeldia* genera (Liu *et al.*, 2014; Parkin *et al.*, 2014; Jeong *et al.*, 2016; Belser *et al.*, 2018; Perumal *et al.*, 2020; Guo *et al.*, 2021; Cho *et al.*, 2022; Xu *et al.*, 2022, 2023; Guerreiro *et al.*, 2023; Yang *et al.*, 2023; Zhang *et al.*, 2023). We also included the *H. incana* HIR1 accession and two other species that are closest to *H. incana* for which draft genome sequences are available, *Brassica tournefortii* and a *Sinapis* sp. (formerly labelled as the *H. incana* HIR3 accession) (Guerreiro *et al.*, 2023). In our phylogenetic tree, all *Brassica* A and C genome types formed a monophyletic clade that represents the *Rapa/Oleracea* clade of the Brassiceae, while all other B genome types (i.e. *B. nigra* and *S. arvensis*) formed another monophyletic clade. This is consistent with previous studies which suggested a close relationship between *B. rapa* and *B. oleracea* (Cheng *et al.*, 2014), and between *B. nigra* and *S. arvensis* (Yang *et al.*, 2023). All *H. incana* accessions were grouped closely to *Sinapis* sp., which hereafter we refer to collectively as the H genome type. Overall, our results support *R. sativus* and *H. incana* being closer to the *Brassica* B genome type than to the A/C type. Notably, our tree topology is consistent with that of Cho *et al.* (2022) regarding the placement of *R. sativus* and *Brassica* species and with that of Garassino *et al.* (2022) regarding the placement of *H. incana* and *Brassica* species. However, our tree topology is different from the tree constructed by Yang *et al.* (2023), in which *R. sativus* is grouped together with the *Brassica* A/C genome type, and inconsistent with the tree topology proposed by Huang *et al.* (2016), in which *R. sativus* and *H. incana* are grouped together with the *Brassica* A genome type. These incongruencies among nuclear species trees are likely to be the result of the differences in gene sets included in the studies, in addition to the methods they used for phylogenetic reconstruction.

We also increased the number of single-copy genes to 5675 by restricting our analysis to a total of five Brassiceae species, selecting one representative genome of each of the three *Brassica* A/C/B types, *R. sativus*, *H. incana*, and outgroup *A. thaliana* (Supplementary Data Table S11), which additionally allowed us to quantify the different gene tree topologies better. The species tree had the same topology as that in Fig. 3A for these species, and the cloudogram of gene tree topologies is shown in Fig. 3B. Aside from the major species tree topology (blue), other topologies were also recovered (green and red), which, interestingly, resembled the discordant topologies found in previous studies (Huang *et al.*, 2016; Yang *et al.*, 2023). We detected more conflicts in nodes involving *B. nigra*, *R. sativus* and *H. incana*, with concordance between 42 and 52 % (Fig. 3B; Supplementary Data Fig. S15). A detailed comprehensive analysis of tree topologies among gene trees derived from these 5675 single-copy genes will be presented and discussed in the next section.

### *Global genome synteny analyses support a potential hybridization or introgression origin of* H. incana *and* R. sativus *from the* Brassica *ancestors*

**Fig. 4.**
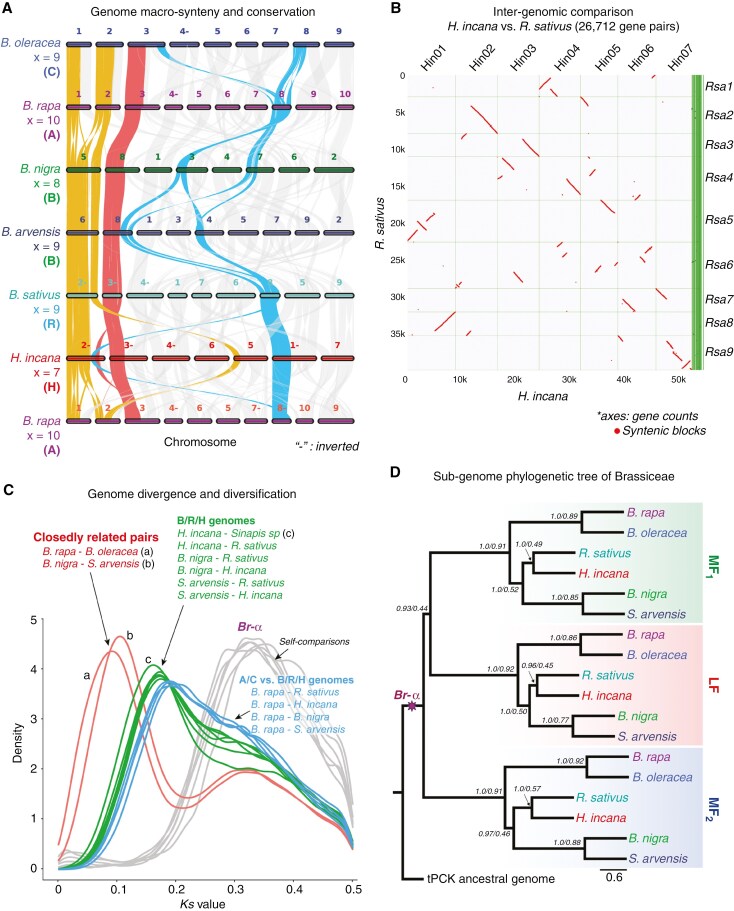
Global genome synteny comparative analyses of *Hirschfeldia incana* and other Brassiceae species. (A) Genome macro-synteny plot of six representative Brassiceae genomes for each group in A, showing intermediate characteristics of *Raphanus sativus* and *H. incana* genomes compared to *Brassica* A/C/B genome types. For example, a conserved syntenic region across all six species (red); a conserved region that is well retained in *Brassica* B genome type, *R. sativus* and *H. incana* (yellow); and a conserved region that is well retained in *Brassica* A/C genome type, *R. sativus* and *H. incana* (blue). Syntenic blocks (*minspan = 30 genes*) were used for each pair-wise comparison among the selected genomes. Chromosome length is not at scale. Chromosome order is rearranged based on the syntenic relationship between two adjacent genomes. ‘-’ denotes inverted sequence. (B) Inter-genomic synteny comparison between the *H. incana* and *R. sativus* genomes. Syntenic blocks (*minspan = 30 genes*) between genomes were aligned. Only true orthologues are shown. Axes show gene count for each genome. (C) Genome divergence among the selected species. *Ks* distributions were coloured for four groups, comparisons among closely related genome pairs (red), among B/R/H genomes (green), among A/C and B/R/H genomes (blue) and self-comparisons of the included genomes (grey). The self-comparison of each highlights the recently shared *Br-α* whole-genome triplication event in these genomes (similar to that in Fig. 2C), while pairwise genome comparisons highlight species divergence. (D) Sub-genome tree of six selected Brassiceae species. The tree was reconstructed using the species-tree approach based on 90 genes located on the tPCK genomic block F found syntenic across all sub-genomes of the six genomes. The tree was rooted using the ancestral tPCK genome as the outgroup. Supporting values at each node are posterior probability and quartet scores, respectively. Branch length represents coalescence units. The genomes of *Brassica rapa* Chiifu, *Brassica oleracea* JZS, *Brassica nigra* NI100, *Sinapis arvensis* XJ1, *R. sativus* NAULB and *H. incana* NIJ were used.

We next analysed global genome synteny among six selected Brassiceae species for which chromosome-level assemblies are available (Fig. 4A). These six genomes formed three pairs that showed a high level of synteny to each other, including *B. rapa* (*x* = 10)–*B. oleracea* (*x* = 9), *B. nigra* (*x* = 8)–*S. arvensis* (*x* = 9) and *H. incana* (*x* = 7)–*R. sativus* (*x* = 9). These represent an array of chromosome numbers that was derived from the ancestral tPCK karyotype (*x* = 7; Lysak *et al.*, 2016; Schranz *et al.*, 2006). In line with expectations from phylogenetic reconstruction, the *H. incana* genome exhibited a greater degree of synteny with the *R. sativus* genome than with any other genomes (Fig. 4A, B; Supplementary Data Fig. S16). We also observed several blocks that are well conserved across the six genomes (i.e. red block), reflecting their common ancestry. Additional support for potential hybridization or introgression comes from the intermediate genome structure of *H. incana* and *R. sativus* in relationship to the *Brassica* A/C and B genome types. For example, a highly syntenic region (yellow) was detected across *B. nigra* [chromosome (chr)5], *S. arvensis* (chr6), *H. incana* (chr2) and *R. sativus* (chr2), whereas it was rearranged in both *B. rapa* and *B. oleracea* genomes (chr1 and chr2) (Fig. 4A). In contrast, another highly syntenic region (blue) is conserved across *B. rapa* (chr8), *B. oleracea* (chr8), *H. incana* (chr1) and *R. sativus* (chr8), whereas it was rearranged in *B. nigra* and *S. arvensis*. These might have a shared evolutionary origin, but their independent origin through breakpoint reuse (Li *et al.*, 2016) is also possible.

By analysing the distribution of *Ks* values of orthologous genes among the selected genomes, we reconstructed their history of lineage divergence after their shared *Br-α* WGT event (Fig. 4C). After divergence from the *Brassica* A/C, the *Brassica* B species was then separated from the *Raphanus* and *Hirschfeldia* species. The smallest *Ks* peaks of comparisons between *B. rapa* and *B. oleracea* (A/C genomes) or between *B. nigra* and *S. arvensis* (B genomes) suggest that they are closer to each other than to any other species, and the split of species in these two pairs occurred more recently. Among all included R/H genomes, *Sinapis* sp. was the closest species to *H. incana*, although their *Ks* peak was only slightly smaller than that of other R vs. H or B vs. R/H genome comparisons.

**Fig. 5.**
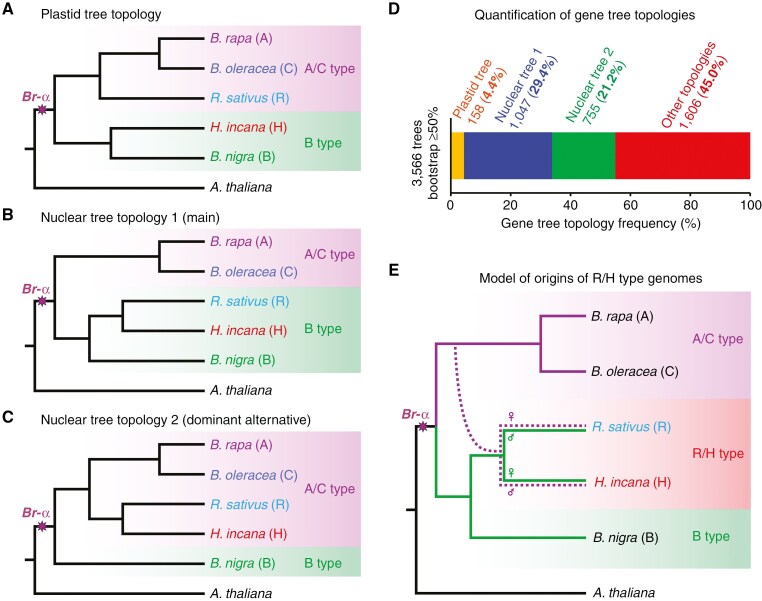
Species tree incongruency and gene tree topology analysis of selected Brassiceae genomes. (A–C) Three focal gene tree topologies that were analysed among the above species including plastid, main nuclear and dominant alternative nuclear topologies, respectively. (D) Quantification of gene tree topologies (in panels A–C) in the subset of 3566 filtered gene trees from the initial set shown in Fig. 3B, keeping only gene trees with bootstrap support ≥ 50 %. (E) A model for the possible origins of the *Raphanus sativus* and *Hirschfeldia incana* genomes deriving from the *Brassica* A/C and B genome types. The maternal origins were determined based on the plastid tree topology (panel A), and the species tree is based on the major nuclear topology (panel B). Dotted line denotes a lower level of hybridization/introgression origin from the *Brassica* A/C, in comparison to that of *Brassica* B genome types. The genomes of *Arabidopsis thaliana* Col-0, *Brassica rapa* Chiifu, *Brassica oleracea* JZS, *Brassica nigra* NI100, *Sinapis arvensis* XJ1, *R. sativus* NAULB and *H. incana* NIJ were used.

Recently, Walden and Schranz (2023) recommended using a synteny-based approach for a more reliable identification of true orthologues for phylogenetic studies. We therefore tested whether this approach could aid in resolving the issues with OrthoFinder single-copy orthologue genes, as discussed earlier. Given that the approach uses all available triplicated gene copies (i.e. multi-copy genes), it could recover both species trees and sub-genome trees using the same set of markers. For this analysis, we focused on 90 syntenic orthologous genes located on the ancestral tPCK genomic block F that were retained in triplicate across three sub-genomes of six selected Brassiceae species (Supplementary Data Table S12; Fig. S17). Both sub-genome and species trees derived from these 90 selected syntenic orthologues (Fig. 4D; Supplementary Data Fig. S18) are consistent with our previous species tree based on OrthoFinder single-copy orthologues (Fig. 3A) and with the main nuclear topology (Figs 3B and 5B). In the sub-genome tree, all sub-genome types across species were shown to be more similar to each other than different sub-genome types within the same species. Each sub-genome type formed a monophyletic clade, with the LF and MF_1_ sub-genomes being grouped closer to each other than either to the MF_2_ sub-genome. The results indicate that a synteny-based approach could be used as a reliable method for species tree inference.

### *Intermediate characteristics of* H. incana *and* R. sativus *explain their incongruent phylogenetic placement in relationship to the* Brassica *species*

Based on the plastid phylogeny, *R. sativus* and the *Brassica* A/C species belong to the *Rapa/Oleracea* clade, whereas *H. incana* and *Brassica* B species belong to the *Nigra* clade within the Brassiceae tribe (Arias and Pires, 2018; Guerreiro *et al.*, 2023). In contrast, our nuclear phylogeny (Fig. 3A) recovered a tree topology in which both species are grouped closer to the *Brassica* B species than to the A/C type species. This cyto-nuclear discordance might indicate past hybridization or introgression from an ancestor of the A/C/B clades to *R. sativus* and *H. incana*. Because their genome structures also suggest that *R. sativus* and *H. incana* exhibit intermediate characteristics between *Brassica* A/C and B genome types, we wondered whether hybridization/introgression could explain the discrepancy in the nuclear phylogenetic placement of these species among published studies. Therefore, following the topology quantification approach of Forsythe *et al.* (2020), we counted gene trees among the 5675 single-copy genes in the previous cloudogram (Fig. 3B) based on three topologies that represent the plastid topology (Fig. 5A) and the two most dominant nuclear topologies (Fig. 5B, C) corresponding to two major species trees in published studies. Our results (Fig. 5D) revealed that among 3566 filtered single-copy gene trees (bootstrap support ≥ 50 %), the most common topology (29.4 %) supported *R. sativus/H. incana* being closer to the *Brassica* B genome (nuclear topology 1), whereas 21.2 % of trees supported them being closer to A/C type (nuclear topology 2). Only 4.4 % of gene trees followed the plastid topology, which could be those nuclear genes that shared the evolutionary history of the chloroplast genes as a result of selection for cyto-nuclear compatibility (Forsythe *et al.*, 2020). The result suggests that, although these are single-copy genes identified across a set of selected genomes, different published studies might have used different gene subsets influenced by the number of genomes included (in addition to different tree reconstruction methods), which could, in turn, affect the phylogenetic inference. This might explain the incongruency observed in published species trees regarding the placement of *R. sativus* and *H. incana* in relationship to the species within the *Brassica* ‘triangle of U’.

Another potential factor that might affect the species tree topologies based on single-copy orthologous genes is that these genes could have originated from different sub-genomes owing to differential gene loss rates among sub-genomes (Cheng *et al.*, 2014; Smith and Hahn, 2021; Xiong *et al.*, 2022). Indeed, in the 5675 single-copy orthologues in the *H. incana* genome, we found that ~46.7 % are located on the LF sub-genomes, while 24.7 and 15.6 % are on MF_1_ or MF_2_, respectively (Supplementary Data Table S13). This suggests that, for genomes that underwent WGD/WGT events followed by rediploidization, single-copy paralogues from different sub-genomes, if taken as orthologues, could potentially contribute to nuclear species tree discrepancy. More specifically, a significant proportion of these single-copy orthologues could be pseudo-orthologues instead of true orthologues, which could be problematic for species tree inference. Interestingly, when comparing our single-copy genes with the sets of single-copy loci commonly used in phylogenomics (B764, Brassicaceae-specific dataset: Nikolov *et al.*, 2019; Hendriks *et al.*, 2023; and A353, Angiosperms universal bait set: Johnson *et al.*, 2019), we found that a higher proportion of genes (55 %) was from the *H. incana* LF sub-genome, potentially owing to the more conserved nature of loci that are single-copy across a larger evolutionary time frame. It is important to note that, although we focused on two main potential factors, hybridization/introgression and single-copy pseudo-orthologues, our analysis cannot rule out that incomplete lineage sorting could be an alternative hypothesis and additional factor to explain the species tree incongruency.

Collectively, our new phylogenetic analyses based on a large single-copy gene sets and multi-copy syntenic orthologues consistently recovered a tree topology in which *R. sativus* and *H. incana* (R/H genome types) were placed closer to the *Brassica* B genome type than to the A/C genome types (Figs 3 and 4D). However, we showed that hybridization or introgression might have contributed to the presence of genes displaying alternative tree topologies. In particular, in the light of the observed cytonuclear discordance, with *R. sativus* grouping with the *Brassica* A/C genome clade in plastid phylogenies, introgression is a likely scenario, at least for this species, while the intermediate genome structure of both R/H genomes suggests that hybridization might have played a role in the evolutionary past of *H. incana*. Additionally, we also found low quartet support and conflicting gene tree topologies among single-copy genes (Figs 3B and 5A–C). These might be the result of differential gene loss during the process of gene rediploidization following the Brassiceae-specific *Br-α* WGT event. Consequently, based on our phylogenetic and genome structure evidence, we propose a model of evolutionary history of the R/H genome types from the *Brassica* B ancestors (Fig. 5E), followed by lower levels of introgression from the *Brassica* A/C genome type ancestors. During this process, *R. sativus* obtained and retained the A/C plastid type, whereas *H. incana* kept the B plastid type.

### Whole-genome triplication-retained genes that show a sub-genome expression bias are associated with distinct biological processes

In comparison to its Brassiceae relatives, the C_3_ species *H. incana* was reported to display high photosynthesis rates in high-light conditions (Canvin *et al.*, 1980; Garassino *et al.*, 2022) and the ability to grow in lead contaminated soils (Auguy *et al.*, 2013; Hasnaoui *et al.*, 2022). We hypothesized that this was a result of differential gene retention of WGT gene copies, possibly in a sub-genome-biased fashion, followed by neo-/sub-functionalization in the *H. incana* genome that gave rise to its ability to accumulate biomass and thrive in such conditions. To provide a comprehensive analysis of triplicated genes in the *H. incana* genome and their potential associations with its adaptive evolution of high-photosynthesis traits in high-light conditions, we studied three gene categories: triad, dyad and single-copy genes. These loci are those which respectively retained three, two or one homologous gene copy of the triplicates from the ancestral tPCK copy after the *Br-α* WGT event.

Initially, using sub-genome information in Fig. 2D, we identified a total of 2103 triads and 6457 dyads. The number of triads identified in *H. incana* is similar to that of other Brassiceae genomes reported by Yang *et al.* (2023), ranging from 1531 to 2183. GO biological process and KEGG pathway analyses of the 2103 triads and 6457 dyads showed an enrichment (FDR-corrected *P* ≤ 0.05) for genes related to plant organ development, growth, shoot system development and morphogenesis, responses to hormones and stimuli, and photosynthesis and carbon metabolism (Supplementary Data Figs S19 and S20). Many of these enriched terms overlap with those from an analysis of upregulated genes in high-light conditions (Supplementary Data Table S14). The results suggest that WGT genes retained for these processes followed by neo-/sub-functionalization could have facilitated the adaptive responses to high-light conditions in *H. incana*.

**Fig. 6.**
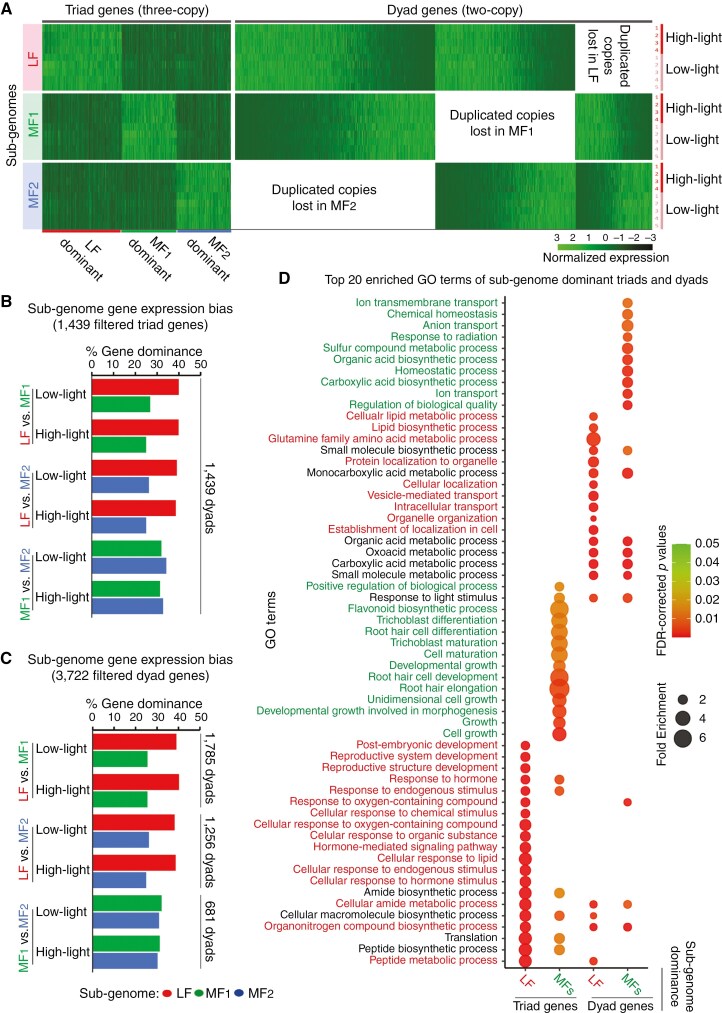
Analysis of sub-genome biased expression of WGT triad and dyad gene copies in the *Hirschfeldia incana* genome. (A) Gene expression of the identified triads (three syntenic triplicated copies) and dyads (two syntenic copies) in two conditions, low-light (five replicates) and high-light (four replicates), taken from whole canopy transcriptomes in the study by Garassino *et al.* (2024). Gene expression data are presented based on sub-genomes and sorted based on the expression levels across the three sub-genomes. For each gene (column), the expression data were column-normalized across all samples, after excluding columns with sum of gene expression of zero in the initial sets. (B, C) Gene expression dominance in different *H. incana* sub-genome comparisons, LF–MF_1_, LF–MF_2_ and MF_1_–MF_2_, calculated for 1439 triad and 3722 dyad gene sets obtained from the initial 2103 and 6457 respective gene sets in A after filtering out low-/non-expressed genes. Gene expression dominance between a pair of duplicated genes was determined using a threshold |fold-change| ≥ 2, *P* ≤ 0.05. The numbers of genes used are indicated next to the bar charts. (D) Gene ontology (GO) enrichment analysis of triad and dyad genes that showed dominance in the LF and MF_1_/MF_2_ sub-genomes, respectively. Top 20 most significant GO terms (false-discovery rate-corrected *P* ≤ 0.05) for each gene set are shown. For GO enrichment of the total 2103 triad and 6457 dyad genes, see Supplementary Data Figs S19 and S20.

Garassino *et al.* (2024) generated transcriptome data of *H. incana* whole canopies from plants grown in contrasting low-light and high-light conditions (Supplementary Data Fig. S21). We used these data to analyse gene expression patterns of the identified triad and dyad genes, as shown in Fig. 6A. To test whether there is an expression bias among the identified triads originating from different sub-genomes, we compared pairwise expression patterns (LF vs. MF_1_, LF vs. MF_2_, and MF_1_ vs. MF_2_) using the method outlined by Cheng *et al.* (2012) and a threshold |fold-change| ≥ 2, *P* ≤ 0.05. Of the total 2103 triads, we further filtered out lowly expressed genes to obtain 1439 triads that had a reliable expression level for cross-sub-genome comparisons. Of these, we found that ~40 % of triad genes showed dominance in the LF compared with MF_1_ sub-genome in low- and high-light conditions, whereas it was only 25–27 % of that in the MF_1_ more dominant over the LF sub-genome (Fig. 6B; Supplementary Data Table S15). Likewise, when comparing between the LF and MF_2_ sub-genomes, 39 and 25–26 % of genes showed dominance for the respective sub-genomes. However, the percentages of dominant genes between the MF_1_ and MF_2_ genomes were more similar, ranging from 31 to 32 % and from 33 to 34 %, respectively. A similar pattern was observed when comparing a total of 3722 filtered dyad genes (Fig. 6C; Supplementary Data Table S15). When all three sub-genomes were compared, it was found that, of the 1439 filtered triads, 49 and 28 % could be classified as dominant in LF and MF_1_/MF_2_ sub-genomes, respectively (Supplementary Data Table S16). The LF-dominant triad genes were most enriched (FDR-corrected *P* ≤ 0.05) for ‘translation’ and many GO terms related to ‘response to hormones/endogenous stimuli’ and ‘reproduction’, whereas the MF_1_/MF_2_-dominant genes were most enriched for ‘cell growth’, ‘developmental growth involved in morphogenesis’, ‘developmental growth’, ‘flavonoid biosynthesis process’ and ‘response to light stimuli’ (Fig. 6D; Supplementary Data Table S16). Among the 3722 filtered dyads, apart from shared enriched terms related to ‘response to light stimuli’ and ‘carboxylic acid metabolic’, the LF-dominant dyad genes were most enriched for terms related to ‘organelle organization’ and ‘protein localization to organelle’, whereas MF_1_/MF_2_-dominant genes were enriched for ‘homeostasis’ and ‘ion transport’ processes (Fig. 6D; Supplementary Data Table S16).

Although we tested sub-genome gene expression bias only in leaf tissues grown in two conditions in *H. incana*, the results are consistent with the observations in other Brassiceae genomes (Cheng *et al.*, 2012; Yang *et al.*, 2023). There might be different gene copies within each triad and dyad that are expressed in a tissue-specific manner. However, this sub-genome expression bias was also reported in different tissues; for example, in leaf, stem and root tissues of *B. rapa* (Cheng *et al.*, 2012), or in leaf and stem tissues of six Brassiceae genomes (Yang *et al.*, 2023). Altogether, our results indicate a bias in sub-genome gene retention and expression (i.e. LF more dominant over MF_1_/MF_2_) in the *H. incana* genome. Genes that showed a sub-genome expression dominance appeared to be associated with different biological processes. More specifically, the LF-dominant genes were most enriched for terms related to responses to stimuli/hormones and organelle organization, whereas MF_1_/MF_2_-dominant genes were most enriched for growth/development and photosynthesis-related terms.

Next, we focused on a total of 6769 single-copy genes that we could confidently assign to the three sub-genomes, of which 3527 (52 %) were found on the LF, 1932 (29 %) on the MF_1_ and 1310 (19 %) on the MF_2_ sub-genome, respectively. These genes were found to be single copy in both *A. thaliana* and *H. incana* genomes and were derived from a total of 7952 originally identified in our OrthoFinder analysis. Interestingly, GO enrichment analysis of these gene sets suggested an enrichment (FDR-corrected *P* ≤ 0.05) for several organelle-related terms, ‘RNA modification’, ‘chromosome segregation’, ‘embryo development’, ‘cell cycle’ and ‘reproduction’ within single-copy genes from the LF sub-genome; ‘mismatch repair’, ‘reciprocal meiotic/homologous recombination’ and ‘plastid organization’ within those from MF_1_; and ‘replication fork processing’, ‘DNA replication maintenance’, ‘response to DNA damage stimulus’, ‘DNA repair’, ‘RNA modification’ and ‘DNA metabolic process’ within those from the MF_2_ sub-genome (Supplementary Data Fig. S22). The result is consistent with findings in previous studies that, unlike genes related to transcription factors, ribosomal proteins and kinases, genes related to DNA repair and organelle-targeted pathways tend to return to single copy after the WGD/WGT event (De Smet *et al.*, 2013). This result highlights the importance of studying the mechanisms that lead to the sub-genome-biased retention of single-copy genes, because many chloroplast-targeted genes are single copy and involved in the photosynthesis machinery.

Evidence is accumulating that polyploidy could potentially aid plants in adapting to (and thriving in) new challenging environments and stressful climates (Van de Peer *et al.*, 2017; Stevens *et al.*, 2020). For example, Feng *et al.* (2024) found a strong selection on three-copy retained gene families associated with adaptive response to new environments in the mangrove tree (*Sonneratia alba*). These include genes related to root development and salt tolerance that enhance plant adaptive response to intertidal zones. In relationship to photosynthesis traits, several studies, including those by Wang *et al.* (2009*b*) and Hoang *et al.* (2023), showed the contribution of WGD to the evolution of C_4_ photosynthesis from the C_3_ ancestral state in two evolutionarily distant families, Poaceae and Cleomaceae, respectively. Our results on retained WGT genes and their sub-genome dominance, especially those related to plant response to endogenous stimuli, morphogenesis, development, organelle organization, chloroplast-targeted pathways and photosynthesis, might reflect important gene families that were involved in the evolution that led to the high-photosynthesis traits at high light intensity in *H. incana*.

### *Analysis of gene families related to leaf physio-biochemical–anatomical changes that potentially facilitate adaptation to high light intensity in* H. incana

**Fig. 7.**
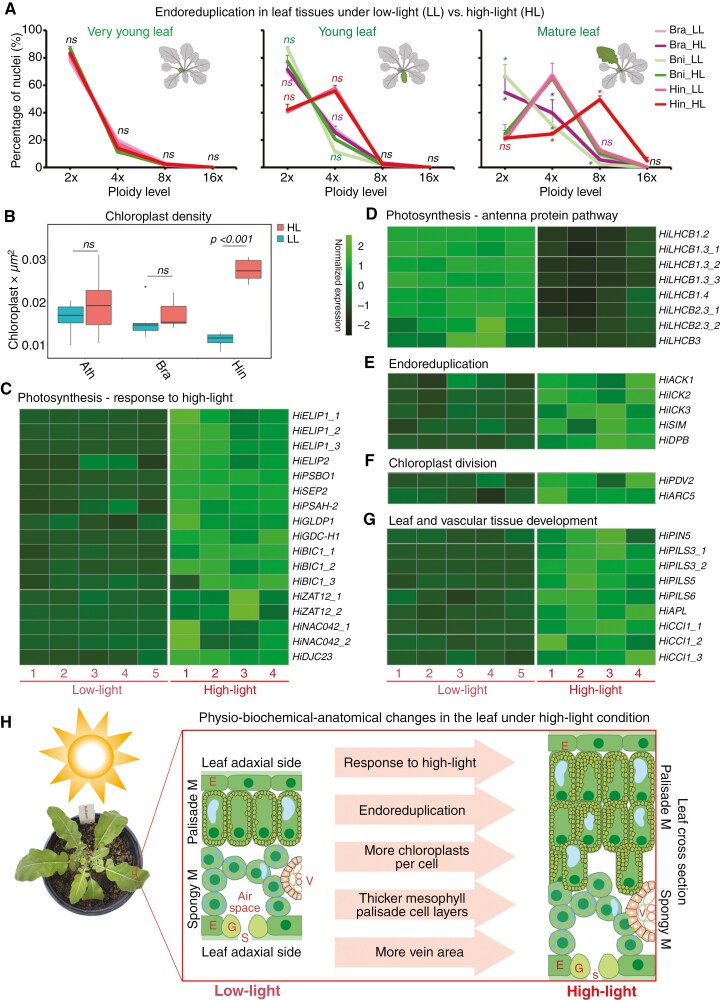
Analysis of gene families associated with adaptive evolution to high-light conditions in the *Hirschfeldia incana* genome. (A) Endoreduplication level of plants grown under low-light and high-light conditions measured by flow cytometry. For each time point, three samples were used. Data are presented as the mean ± s.e.m. Error bars indicate s.e.m. *ns*, non-significant. *P ≤ 0.05 by Student’s *t*-test (*N* = 3). (B) Chloroplast density of plants grown in low-light and high-light conditions. Statistical significance was assessed by Student’s *t*-test (*N* ≥ 5 for each sample). (C–G) Expression analysis of gene families associated with physio-biochemical–anatomical adaptive changes that are likely to facilitate the high photosynthesis in *H. incana*, including genes related photosynthesis, endoreduplication, chloroplast division, leaf and vascular development. Only genes that showed an upregulation (false-discovery rate-corrected *P* ≤ 0.05 and |fold-change| ≥ 2) in the whole canopy transcriptomes of low- and high-light conditions (Garassino *et al.*, 2024) were included. Gene expression data are TPM values and were row-normalized (per gene). (H) A model of *H. incana* leaf physio-biochemical–anatomical adaptive changes in response to high-light conditions. Abbreviations: Ath, *Arabidopsis thaliana*; Bra, *Brassica rapa*; Bni, *Brassica nigra*; E, epidermis; G, guard cells; Hin/Hi, *Hirschfeldia incana*; HL, high light; LL, low light; M, mesophyll; S, stomata; V, vasculature.

Given that our previous analyses suggested several gene groups that are related to adaptive changes that might explain the high-photosynthesis traits in *H. incana* and its ability to withstand high-light conditions, our next focus was particularly on the expression of genes involved in the key changes found in the plants grown under high light. These key changes were based upon the recent findings on the significant physiological, biochemical and anatomical differences in the *H. incana* leaf tissues in response to high light compared with its relatives. More specifically, the changes consist of: (1) a higher average gross CO_2_ assimilation rate at high irradiance (Garassino *et al.*, 2022); (2) a higher endoreduplication (endopolyploidy) level (Fig. 7A; Supplementary Data Table S17); (3) a smaller antenna size and more chloroplasts of smaller size (Caracciolo *et al.*, 2024; Fig. 7B); and (4) the development of thicker multilayer palisade mesophyll cells, a higher vasculature volume and mesophyll surface area (Retta *et al.*, 2024). Among these, to our surprise, when compared with *B. rapa* and *B. nigra*, *H. incana* showed a much higher endoreduplication level in mature leaves under high light (50 vs. ≤10 % nuclei at 8× ploidy level; Fig. 7A). Our hypothesis was that these changes are the results of differential expression of genes associated with the photosynthesis machinery (i.e. response to high-light stimuli and photosystem proteins), cell division (i.e. cell cycle, endoreduplication and plastid division) and leaf development (mesophyll formation, vein development and hormone signalling pathways). To this end, we identified genes differentially expressed between low-light and high-light conditions in the whole canopy transcriptome data of *H. incana* (Garassino *et al.*, 2024) using our updated gene models. A total of 2012 up- and 2126 downregulated genes were found using a threshold FDR-corrected *P*-value ≤ 0.05 and |fold-change| ≥ 2 (for a list, see Supplementary Data Table S18 and for GO enrichment analysis, see Supplementary Data Table S14).

Interestingly, among the upregulated genes related to photosynthesis and response to high light intensity (Fig. 7C, D), several genes encoding for transcription factors and proteins were found, including *ELIPs* (*EARLY LIGHT-INDUCIBLE PROTEINs*), *BIC2* (*BLUE-LIGHT INHIBITOR OF CRYPTOCHROMES 2*), *ZAT12/RHL41* (*RESPONSIVE TO HIGH LIGHT 41*), *NAC042* (*NAC DOMAIN CONTAINING PROTEIN 42*) and *DJC23* (*DNA J PROTEIN C23*). Besides ELIPs that are known to have roles in photoprotection and chlorophyll biosynthesis (Hutin *et al.*, 2003), these might be genes that contribute to the adaptation to the high-light conditions and facilitate the high photosynthetic efficiency in *H. incana*. For example, ZAT12 was shown to play a central role in high-light acclimation and response to oxidative stress in *A. thaliana* (Davletova *et al.*, 2005), while the transcription coactivator BIC1 promotes brassinosteroid signalling and plant growth (Yang *et al.*, 2021). NAC042, a reactive oxygen species-responsive transcription factor, enhances stress tolerance and delays senescence (Wu *et al.*, 2012), and DJC23 is involved in optimizing photosynthetic reactions (Chen *et al.*, 2010). Additionally, we noticed that most of the genes encoding photosystem II LHCBs (LIGHT HARVESTING CHLOROPHYLL A/B-BINDING PROTEINs) and photosystem I LHCAs (PHOTOSYSTEM I LIGHT HARVESTING COMPLEX PROTEINs) were, to different extents, suppressed by high-light conditions. However, we found only *LHCBs* genes (i.e. *LHCB1.2*, *LHCB1.3s*, *LHCB1.4*, *LHCB2.3s* and *LHCB3*) to be significantly downregulated (Fig. 7D). This might explain the reduction of functional antenna size of photosystem II but not that of photosystem I, as observed by Caracciolo *et al.* (2024).

Among the upregulated genes related to cell division, we found several interesting genes associated with cell cycle control [‘CYCLIN-dependent protein kinase inhibitor activities (CKI)’; Fig. 7E] that might be responsible for the high endoreduplication level by inhibiting cell division (Vieira *et al.*, 2014). These include *ACK1* (*ARABIDOPSIS CDK INHIBITOR 1*), *ICKs* (*KIP-RELATED PROTEIN*), *SIM* (*SIAMESE*) and *DPP* (*DP* protein). It was found that plants could sustain growth in different potentially stressful conditions (e.g. high ultraviolet-B irradiation) by endoreduplication, which is a result of one or more rounds of genome replication without mitosis (Scholes and Paige, 2015; Zedek *et al.*, 2020). The upregulation of these cell division inhibitor genes might be related to the high endoreduplication level found in *H. incana* mature leaves in high-light conditions. Additionally, there were two genes involved in plastid division, *PDV2* (*PLASTID DIVISION2*) and *ARC5* (*ACCUMULATION AND REPLICATION OF CHLOROPLAST 5*; Fig. 7F). PDV1 and PDV2 are known to function together with ARC5 in the chloroplast to mediate chloroplast division (Miyagishima *et al.*, 2006). Among genes related to leaf and vascular system development (Fig. 7G), *PIN5* (*PIN-FORMED 5*), several *PILS* (*PIN-LIKES*) *3*, *5* and *6*, *APL* (*ALTERED PHLOEM DEVELOPMENT*) and *CCI1* (*CLAVATA COMPLEX INTERACTOR 1*) were upregulated in high-light samples. PIN5 and PILS were shown to be involved in auxin transport pathways controlling vein patterning (Mravec *et al.*, 2009; Sawchuk *et al.*, 2013) and auxin signalling during environmental stimuli-induced growth adaptation (Waidmann *et al.*, 2023). APL is a transcription factor that regulates vascular tissue development in *Arabidopsis* (Bonke *et al.*, 2003), and CCI1 is involved in the WUSCHELL/CLAVATA signalling pathway functioning in shoot meristem development (Gish *et al.*, 2013).

Overall, our gene expression analysis supports the key physiological, biochemical and anatomical adaptive changes observed in the *H. incana* leaf tissues in response to high light. Given that the transcriptome data were derived from one time point of the whole canopy (i.e. an averaged sample of different developmental stages), our analysis might have missed important genes that were expressed in a more tissue or cell type-specific or developmental stage-specific manner (i.e. transcription factor genes, hence diluted in a bulk sample). In this case, transcriptomes of dissected tissues or single cells or time series might be needed to identify key players in these processes. Additionally, our analysis was based solely on transcriptional evidence that supports the observed phenotypic changes, and further proteomic experiments might need to be conducted to validate this and to identify other genes involved in these changes that were not detected in the transcriptomic analysis. Finally, based on the evidence from comparative phenotypic, genomic and transcriptomic analyses, we propose a model of key processes related to physio-biochemical–anatomical changes in the leaf of *H. incana* in response to high light that might have facilitated its high-photosynthesis traits (Fig. 7H).

### Conclusions

In summary, we successfully reconstructed an improved chromosome-level genome assembly of *H. incana* (NIJ accession, v.2.0) based on a combination of ONT sequencing and Hi-C technologies. The improved *H. incana* genome assembly and annotation enabled us to elucidate the WGT history of the *H. incana* genome from the common *Brassica* tPCK ancestor, and genome evolution of this species in relationship to other species within the Brassiceae tribe. We were able to assign the triplicated ancestral genomic blocks within the *H. incana* genome into three sub-genomes, with the LF sub-genome showing dominance in gene retention in addition to gene expression over the MF_1_/MF_2_ sub-genomes. This sub-genome expression divergence among WGT retained gene copies is likely to be attributable to the neo-/sub-functionalization processes. The *H. incana* genome appears to be similar to the *R. sativus* genome and displays intermediate characteristics of *Brassica* A/C and B genome types. This result might explain the discrepancy observed in the published studies regarding their phylogenetic placement in relationship to the ‘triangle of U’ species. Using the information obtained from comparative physio-biochemical and anatomical studies as a guide, we illustrated the expression changes of the associated gene families which are likely to facilitate the high-photosynthesis traits under high light in *H. incana*. Overall, the improved genome assembly, annotation and results presented in this work will be a valuable resource for future research to unravel the genetic basis of this exceptional species in terms of light-use efficiency and improvement in photosynthesis for enhanced agricultural production.

## SUPPLEMENTARY DATA

Supplementary data are available at *Annals of Botany* online and consist of the following.

Figure S1: Summary of sequencing read data quality used in this study. Figure S2: Size estimation and assembly scheme of the *H. incana* genome. Figure S3: comparison among the assemblies from the Brassiceae. Figure S4: comparison of different annotation approaches of the *Hirschfeldia incana* genome v.1.5. Figure S5: comparison of annotated proteomes and coding sequences (CDS) from selected Brassicaceae genomes. Figure S6: dotplot showing synteny between *Hirschfeldia incana* v.1.0 and v.2.0, analysed by SynMap program. Figure S7: OMArk proteome assessment of selected Brassicaceae genomes. Figure S8: macro-synteny between *Arabidopsis thaliana* and *Hirschfeldia incana*. Figure S9: intra-genomic syntenic (self-comparison) dotplot the *Hirschfeldia incana* genome v.2.0. Figure S10: macro-synteny between *Brassica rapa* and *Hirschfeldia incana*. Figure S11: gene duplication modes of the *Hirschfeldia incana* genome v.2.0 compared with other Brassicaceae genomes. Figure S12: syntenic relationship between the *Hirschfeldia incana* genome v.2.0 and the Brassica tPCK ancestral genomic blocks (*Bra*-ACK). Figure S13: additional plot showing syntenic relationship between the *Hirschfeldia incana* genome v.2.0 and the Brassica tPCK ancestral genomic blocks (*Bra*-ACK). Figure S14: evolutionary history and genome rearrangement estimation of the six Brassiceae genomes. Figure S15: PhyParts tree topology quantification. Figure S16: genome syntenic dotplot and ribbons between *Raphanus sativus* and *Hirschfeldia incana* v.2.0. Figure S17: the identification of the tPCK ancestral genomic block F in the six Brassiceae genomes. Figure S18: species tree reconstructed from 90 triad gene sets from three sub-genomes of each Brassiceae species. Figure S19: GO biological process (upper panel) and KEGG pathway (lower panel) enrichment analyses of all well-retained 2103 triad genes. Figure S20: GO biological process (upper panel) and KEGG pathway (lower panel) enrichment analyses of two-copy retained 6457 dyad genes. Figure S21: a summary of *Hirschfeldia incana* whole canopy transcriptome data. Figure S22: GO biological process enrichment analyses of 3527, 1932 and 1310 single-copy genes found on the LF, MF1 and MF2 sub-genomes. Table S1: sequencing data information for the assembly of the *Hirschfeldia incana* genome v.2.0. Table S2: comparison of assemblies by QUAST. Table S3: mapping rates of Illumina data onto the three assemblies. Table S4: BUSCO completeness of the three *Hirschfeldia incana* assemblies. Table S5: repeat masking of the *Hirschfeldia incana* genome v.2.0. Table S6: BUSCO completeness assessment of the final *Hirschfeldia incana* genome annotation v.2.0 (lifted from v.1.5). Table S7: summary of functional annotation of the *Hirschfeldia incana* gene set. Table S8: assessment of selected proteomes from Brassicaceae by OMArk. Table S9: OrthoFinder orthologue groups clustering results. Table S10: intervals and boundaries of the 26 ancestral genomic blocks in the *Hirschfeldia incana* genome. Table S11: a list of 5675 single-copy genes identified among the selected genomes. Table S12: a set of 90 syntenic orthologous gene groups identified across sub-genomes of six selected Brassiceae species. Table S13: sub-genome assignment of 5675 single-copy genes identified the *Hirschfeldia incana* genome. Table S14: KEGG pathway and GO term enrichment of upregulated genes in high-light compared to low-light condition in *Hirschfeldia incana*. Table S15: sub-genome biased gene expression analysis (pairwise comparisons). Table S16: sub-genome biased gene expression analysis (all three sub-genome comparison). Table S17: endoreduplication analysis of *Brassica rapa*, *B. nigra* and *Hirschfeldia incana* under low-light and high-light conditions. Table S18: a list of 2012 up- and 2126 downregulated genes identified in whole canopy transcriptome data.

mcae179_suppl_Supplementary_Figures_S1-S24

mcae179_suppl_Supplementary_Tables_S1-S18

## Data Availability

Data supporting the findings in this work are available here and in Supplementary Data files. The raw WGS and transcriptome data from Illumina and Nanopore platforms used for this study have been deposited at NCBI SRA database under BioProject PRJNA1045848. The final assembly and annotation (v.2.0) of the *H. incana* genome can be downloaded from Figshare at https://doi.org/10.6084/m9.figshare.25574799. The final genome sequence has also been deposited at DDBJ/ENA/GenBank under the accession JBAWST000000000. The version described in this paper is version JBAWST010000000. Sequence alignment files and machine-readable phylogenetic trees related to phylogenetic analyses presented in this work are available via Figshare at https://doi.org/10.6084/m9.figshare.25574811. The *A. thaliana* araport11 genome was downloaded from Phytozome 13 (https://phytozome-next.jgi.doe.gov/info/Athaliana_Araport11). The *T. parvula* genome v.8.4 was downloaded from http://thellungiella.org. The *B. rapa* Chiifu genome (v.4.0 and v.4.1), *B. oleracea* JZS genome v.2.0 and Broccoli HDEM data were downloaded from the Brassicaceae Database (http://brassicadb.cn/#/). The *B. nigra* NI100 v.2.0 and C2 genomes were downloaded from the Crucifer Genome Initiative https://cruciferseq.ca. The *B. rapa* chinensis Pakchoi genome was obtained from https://doi.org/10.6084/m9.figshare.19589524.v2. The *B. oleracea* genomes for accessions Cauliflower Korso and Kale were respectively downloaded from the Brassica oleracea Genome Database http://www.bogdb.com/genome/cauliflower and Ensembl Genomes https://ftp.ensemblgenomes.ebi.ac.uk/pub/plants/release-58/. The *R. sativus* genomes for accessions WK10039 (v.1.0 and v.2.0) and NAULB were respectively obtained from https://doi.org/10.6084/m9.figshare.21671201.v2, https://www.ncbi.nlm.nih.gov/datasets/genome/GCF_000801105.2/, https://download.cncb.ac.cn/gwh/Plants/Raphanus_sativus_NAU-LB_GWHCBIT00000000. The genomes of *B. nigra* Giebra and *S. arvensis* were obtained from https://doi.org/10.6084/m9.figshare.21442935.v1. The genomes of *B. tournefortii*, *Sinapis* sp. HIR3, *H. incana* HIR1 were obtained from https://doi.org/10.6084/m9.figshare.21671201.v2.
