# Correction to: Expanding the triangle of U: comparative analysis of the *Hirschfeldia incana* genome provides insights into chromosomal evolution, phylogenomics and high photosynthesis-related traits

**DOI:** 10.1093/aob/mcaf020

**Published:** 2025-02-13

This is a correction to: Nam V Hoang, Nora Walden, Ludovico Caracciolo, Sofia Bengoa Luoni, Moges Retta, Run Li, Felicia C Wolters, Tina Woldu, Frank F M Becker, Patrick Verbaarschot, Jeremy Harbinson, Steven M Driever, Paul C Struik, Herbert van Amerongen, Dick de Ridder, Mark G M Aarts, M Eric Schranz, Expanding the triangle of U: comparative analysis of the *Hirschfeldia incana* genome provides insights into chromosomal evolution, phylogenomics and high photosynthesis-related traits, *Annals of Botany*, 2024;, mcae179, https://doi.org/10.1093/aob/mcae179

In the originally published version of the manuscript, there were errors in Table 1 and Figures 2-4. In Table 1, in row for “Number of genes”, column “*Hirschfeldia incana* v.1.0 (NIJ)” numbers should read: “32 313” instead of: ”32 312”. Table 1 should read:

**Table:** 

Genome features	*Brassica rapa* v.4.0 (Chiifu)	*Brassica oleracea* v.2.0 (JZS)	*Brassica nigra* v.2.0 (NI100)	*Hirschfeldia incana* v.1.0 (NIJ)	*Hirschfeldia incana* v.1.5 (NIJ)	*Hirschfeldia iincana* v.2.0 (NIJ)
Chromosome number (*n* = *x*)	10	9	8	7	7	7
Assembled genome size (Mb)	424.59	561.16	506.00	398.50	408.86	408.93
GC content (assembly, %)	37.59	36.75	38.21	36.18	36.20	36.20
Number of scaffolds	10	649	58	384	246	358
Number of pseudomolecules	10	9	8	N/A	N/A	7
Scaffold N50 length (Mb)	43.05	57.88	60.82	5.11	13.76	52.38
Scaffold N90 length (Mb)	30.43	47.84	55.08	0.83	1.99	48.08
Longest scaffold (Mb)	73.37	74.51	70.85	15.00	30.16	63.71
N’s per 100 kb	0.05	26.05	2.47	13.45	1.70	18.08
BUSCO assembly (%)	99.5	99.5	99.2	99.3	99.5	99.2
Number of genes	47 531	59 064	59 852	32 313	54 459	54 457
GC content (main transcript, %)	46.24	46.13	45.92	46.53	46.19	46.19
N50 length (main transcript, bp)	1473	1428	1449	1515	1377	1377
Mean length (main transcript, bp)	1147	1155	1026	1247	1035	1035
BUSCO (main transcripts, %)	97.2	98.7	98.2	96.2	97.8	97.7

instead of:

**Table:** 

Genome features	*Brassica rapa* v.4.0 (Chiifu)	*Brassica oleracea* v.2.0 (JZS)	*Brassica nigra* v.2.0 (NI100)	*Hirschfeldia incana* v.1.0 (NIJ)	*Hirschfeldia incana* v.1.5 (NIJ)	*Hirschfeldia iincana* v.2.0 (NIJ)
Chromosome number (*n* = *x*)	10	9	8	7	7	7
Assembled genome size (Mb)	424.59	561.16	506.00	398.50	408.86	408.93
GC content (assembly, %)	37.59	36.75	38.21	36.18	36.20	36.20
Number of scaffolds	10	649	58	384	246	358
Number of pseudomolecules	10	9	8	N/A	N/A	7
Scaffold N50 length (Mb)	43.05	57.88	60.82	5.11	13.76	52.38
Scaffold N90 length (Mb)	30.43	47.84	55.08	0.83	1.99	48.08
Longest scaffold (Mb)	73.37	74.51	70.85	15.00	30.16	63.71
N’s per 100 kb	0.05	26.05	2.47	13.45	1.70	18.08
BUSCO assembly (%)	99.5	99.5	99.2	99.3	99.5	99.2
Number of genes	47 531	59 064	59 852	32 312	54 459	54 457
GC content (main transcript, %)	46.24	46.13	45.92	46.53	46.19	46.19
N50 length (main transcript, bp)	1473	1428	1449	1515	1377	1377
Mean length (main transcript, bp)	1147	1155	1026	1247	1035	1035
BUSCO (main transcripts, %)	97.2	98.7	98.2	96.2	97.8	97.7

Under subheading “*Nuclear phylogenetic analyses*”, second paragraph, first sentence, text should read: “[…]coding sequences from the 5675 strict single-copy genes’ identified[…]” instead of: “[…]coding sequences from the 5765 strict single-copy genes’ identified[…]”.

In section “RESULTS AND DISCUSSION”, in the fourth paragraph, 3^rd^ sentence, text should read: “Overall, the total number of gene models in genome annotation v.2.0 is higher than that reported for v.1.0 (32 313)[…]” instead of:“Overall, the total number of gene models in genome annotation v.2.0 is higher than that reported for v.1.0 (32 312)[…]”.

In Figure 2, panel C, “*At-“* has been emended to read; ““*At-β”*”.


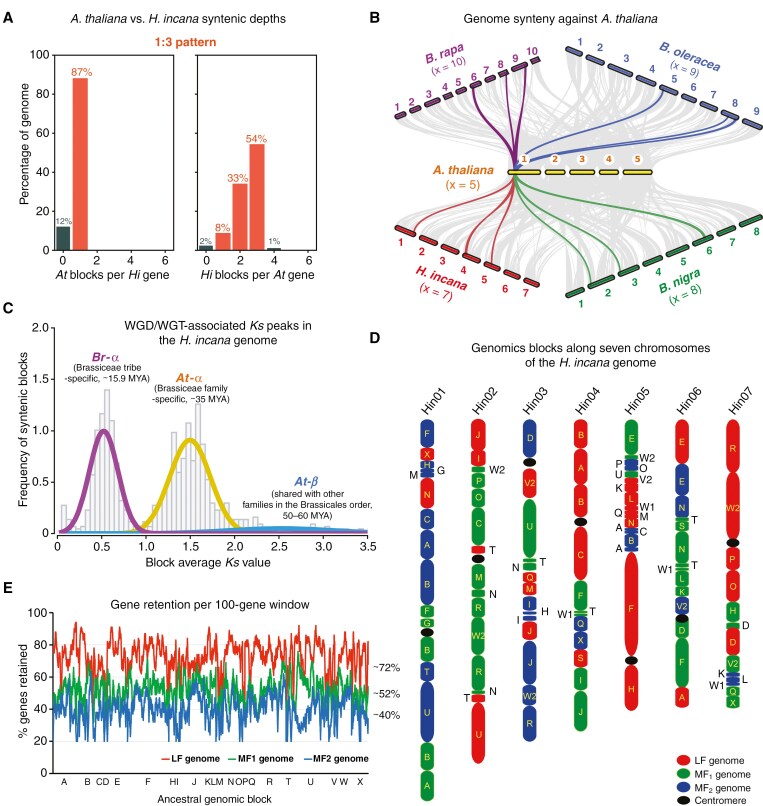


In Figure 3, panel A, “*Sinapsis sp.”* has been emended to read: “*Sinapis sp.”.*


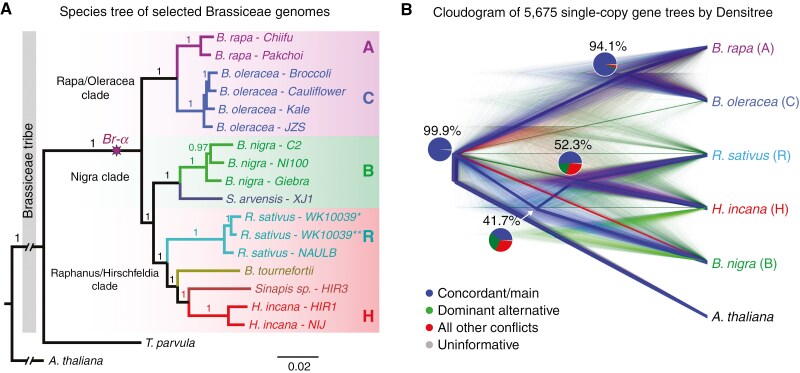


In Figure 4, panel C, “*H. incana - Sinapsis sp* (c)” has been emended to read: “*H. incana* - *Sinapis sp* (c)”; and “*S. arvensis – H. sincana*” has been emended to read: “*S. arvensis – H. incana*”.


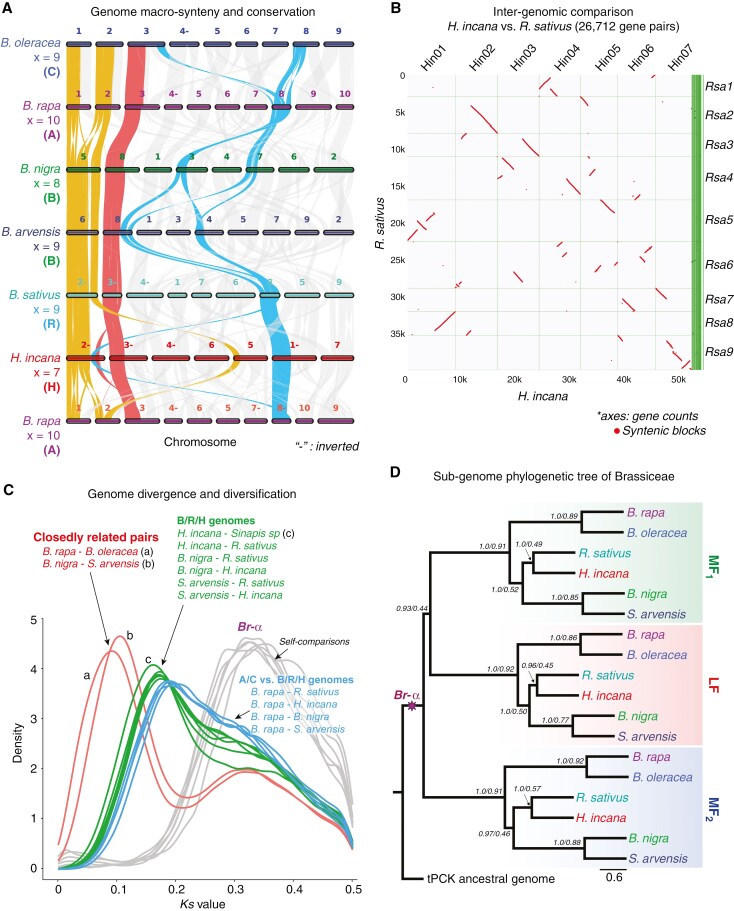


The corrections have been made to the article.

