# Supplementary material for: Expanding the triangle of U: comparative analysis of the Hirschfeldia incana genome provides insights into chromosomal evolution, phylogenomics and high photosynthesis-related traits
Source: Ann Bot. 2024 Oct 24;138(3):758–80. doi: 10.1093/aob/mcae179 (PMC13487439; doi:10.1093/aob/mcae179)
Supplement: Supplementary file 1 [file mcae179_suppl_supplementary_figures_s1-s24.docx]

**SUPPLEMENTARY FIGURES**

**Expanding the Triangle of U: Comparative analysis of the
*Hirschfeldia incana* provides genome insights into chromosomal evolution, phylogenomics and high photosynthesis-related traits**

**Nam V. Hoang^1^,** **Nora Walden^2^_,_ Ludovico Caracciolo^3^, Sofia Bengoa Luoni^4^, Moges Retta^5^,
Run Li^1^, Felicia C. Wolters^1,6^, Tina Woldu^6^, Frank F. M. Becker^4^, Patrick Verbaarschot^1^, Jeremy Harbinson^3^, Steven M. Driever^5^, Paul C. Struik^5^, Herbert van Amerongen^3^,
Dick de Ridder^6^, Mark G.M. Aarts^4^ and M. Eric Schranz^1,*^**

^1^Biosystematics Group, Wageningen University and Research, Droevendaalsesteeg 1, 6708 PB Wageningen, The Netherlands

^2^Centre for Organismal Studies, Heidelberg University, 69120 Heidelberg, Germany

^3^Laboratory of Biophysics, Wageningen University and Research, Stippeneng 4, 6708 WE Wageningen, The Netherlands

^4^Laboratory of Genetics, Wageningen University and Research, Droevendaalsesteeg 1, 6708 PB Wageningen, The Netherlands

^5^Centre for Crop Systems Analysis, Wageningen University and Research, P.O. Box 430, 6700 AK Wageningen, The Netherlands

^6^Bioinformatics Group, Wageningen University and Research, Droevendaalsesteeg 1, 6708 PB Wageningen, The Netherlands

^*^Corresponding author: [eric.schranz@wur.nl](mailto:eric.schranz@wur.nl)

**Figure S1. Summary of sequencing read data quality used in this study.** Quality assessment of a total of ~20 Gb long-read data (N50 = 26 kb, ~48x genome coverage) from combined Nanopore ONT whole-genome sequencing (**A**), and 124 million Hi-C Illumina reads (150 bp, ~44x genome overage) (**B**). (**C-F**) Nanopore RNA sequencing data statistics. (**C**) General read length and quality distribution dot plot. (**D**) Comparison of sequenced sample size in bases bar-chart. (**E**) Comparison of N50 values across samples bar-chart. (**F**) Comparison of sequencing libraries and read length distribution across samples. A 1kb DNA ladder was used. HL and LL denote high-light and low-light, respectively.

**Figure S2**. **Size estimation and assembly scheme of the *H. incana* genome. Upper panel:** Genome size estimation using GenomeScope v2.0 and 102 millions WGS Illumina read data published in Garassino et al. (2022). **Lower panel:** A summary of genome assembly and scaffolding using ONT and Hi-C data for *H. incana.* The genome assembly v1.0 was previously reconstructed based on PacBio SMRT long-read, 10X Genomics linked-read, and Illumina short-read data (Garassino et al., 2022). To symplify, only the assembly of one chromosome is illustrated.

**Figure S3.** **Comparison among the assemblies from the Brassiceae.** Total length comparison (upper panel) and top 15 longest scaffolds (lower panel). In the upper panel: x denotes % of assembly.

**Figure S4. Comparison of different annotation approaches of the *H. incana* genome v1.5.** The genomes of *A. thaliana* Col-0, *B. rapa* Chiifu, *B. nigra* NI100 and *H. incana* v1.0 were used as reference. Annotation with “helixer” in the names indicate those that were reannotated using Helixer (Holst et al., 2023). EVM denotes EvidenceModeler (Haas et al., 2008). FUN indicates Funannotate derived annotation (<https://github.com/nextgenusfs/funannotate>). All FUN annotation sets were obtained from PREDICT step of the pipeline. The final annotation was derived from Hin_v1.5_FUN after the UPDATE step by PASA to add untranslated regions (UTR) data and fix gene models that were not in agreement with the RNA-seq data. This update annotation was then lifted over to genome v2.0 (Hi-C) to obtain the final annotation. *Hin:* *H. incana.*

**Figure S5.** **Comparison of annotated proteomes and coding sequences (CDS) from selected Brassicaceae genomes. (A)** Protein length distribution. **(B)** Cumulative length in percentage of CDS. x denotes % of assembly. **(C)** Cumulative length in Mb of CDS. **(D)** GC content of CDS. Note that the distribution of *H. incana* v1.5 and v2.0 are very similar and largely overlapped.

**Figure S6.** **Dotplot showing synteny between *H. incana* v1.0 and v2.0, analyzed by SynMap program** on the CoGe v7 (<https://genomevolution.org/coge/>) using a syntenic block size minimum = 30 genes. Only “true orthologs” between the two assemblies were used**.**

**Figure S7. OMARK proteome assessment of selected Brassicaceae genomes.** The OMArk v0.3.0 based on 17,999 conserved orthologs of the Brassicaceae family (Nevers et al., 2022) were used to analyze and compare the annotations.

**Figure S8. Macro-synteny between *A. thaliana* and *H. incana*.** The syntenic depth ratio was set to
1 vs. 3 to use only true orthologous genes between the 2 genomes. Plot was generated by MCscan v0.8 (Tang et al., 2008) python version (<https://github.com/tanghaibao/jcvi/wiki/MCscan-(Python-version)>)

**Figure S9. Intra-genomic syntenic (self-comparison) dotplot the *H. incana* genome v2.0.** Plot was generated by MCscan v0.8 (Tang et al., 2008) python version (<https://github.com/tanghaibao/jcvi/wiki/MCscan-(Python-version)>)

**Figure S10. Macro-synteny between *B. rapa* and *H. incana.*** The syntenic depth ratio was set to
1 vs. 1 to use only true orthologous genes between the 2 genomes. Plot was generated by MCscan v0.8 (Tang et al., 2008) python version (<https://github.com/tanghaibao/jcvi/wiki/MCscan-(Python-version)>)

**Figure S11. Gene duplication modes of the *H. incana* genome v2.0 compared with other Brassicaceae genomes.** Modes of duplicated gene copies were analyzed by DupGen_finder (Qiao et al., 2019) with default parameters using the *A. thaliana* genome as reference. *Tpa: T. parvula, Bra: B. rapa, Bol: B. oleracea, Bni: B. nigra, Rsa: R. sativus, Sar: S. arvensis,* and *Hin: H. incana.* Note that *A. thaliana* and *T. parvula* did not experience the WGT *Br-α* event.

**Figure S12. Syntenic relationship between the *H. incana* genome v2.0 and the Brassica tPCK ancestral genomic blocks (Bra-ACK).** For comparison, the analysis was also done for the *B. rapa* genome v4.0. Plot was generated by MCscan v0.8 (Tang et al., 2008) python version (<https://github.com/tanghaibao/jcvi/wiki/MCscan-(Python-version)>)

**Figure S13. Additional plot showing syntenic relationship between the *H. incana* genome v2.0 and the Brassica tPCK ancestral genomic blocks (Bra-ACK).** Same genomes and comparisons as in **Figure S12** were used. The blocks between U and X are V1, V2, W1 and W2.

**Figure S14. Evolutionary history and genome rearrangement estimation of the six Brassiceae genomes.** The genomes of *B. rapa* Chiifu, *B. oleracea* JZS, *B. nigra* NI100, *S. arvensis* XJ1, *R. sativus* NAULB and *H. incana* NIJ were used. The IAGS pipeline (accessed Feb 2024) (Gao et al., 2022) was run based on the orthologous results from OrthoFinder and the non-overlapping syntenic blocks detected by Drimm-Synteny (accessed Feb 2024) (Pham and Pevzner, 2010).

**Figure S15. PhyParts tree topology quantification** using all gene trees (top panel), gene trees with a bootstrap ≥50% (middle panel) and gene trees with a bootstrap ≥85% (bottom panel). The genomes of *A. thaliana* Col-0 (AT), *B. rapa* Chiifu (Bra), *B. oleracea* JZS (Bol), *B. nigra* NI100 (Bni), *R. sativus* NAULB (Rsa) and *H. incana* NIJ (HIN) were used.

**Figure S16.** **Genome syntenic dotplot and ribbons between *R. sativus* and *H. incana* v2.0.** The genomes of *R. sativus* NAULB and *H. incana* NIJ were used. “-“ denotes inverted sequence.

**Figure S17. The identification of the tPCK ancestral genomic block F in the six Brassiceae genomes. Left panel:** three triplicated ancestral genomic block F showing different fractionation rate corresponding to three sub-genome origins, LF, MF_1_ and MF_2_. **Right panel:** locations of the three triplicated ancestral genomic block F on each Brassiceae genome. The genomes of *B. rapa* Chiifu, *B. oleracea* JZS, *B. nigra* NI100, *S. arvensis* XJ1, *R. sativus* NAULB and *H. incana* NIJ were used. The plots were generated by SynMap ran together with FractBias (Joyce et al., 2017) on the CoGe v7. Note that for *B. nigra* genome, the fragment C7-1 (MF_2_) was not fully shown in the left panel.

**Figure S18. Species tree reconstructed from 90 triad gene sets from three sub-genomes of each Brassiceae species** (as shown in **Figure S17**). All posterior probability supporting values are 1 (100%) and not shown. Branch length represents coalescence units.

**Figure S19. GO biological process (upper panel) and KEGG pathway (lower panel) enrichment analyses of all well-retained 2,103 triad genes** highlighting topmost significant GO terms (FDR-corrected *p* ≤ 0.05).

**Figure S20. GO biological process (upper panel) and KEGG pathway (lower panel) enrichment analyses of two-copy retained 6,457 dyad genes** highlighting topmost significant GO terms (FDR-corrected *p* ≤ 0.05).

**Figure S21. A summary of *H. incana* whole canopy transcriptome data.** The *H. incana* RNA-seq sample correlation (upper panel) and the expression of the identified DEGs in two contrasting low (LL) and high-light (HL) conditions (lower panel). Gene expression was row-normalized. HI: *Hirschfeldia incana*.

**Figure S22. GO biological process enrichment analyses of 3,527, 1,932 and 1,310 single-copy genes found on the LF, MF_1_ and MF_2_ sub-genomes**, respectively, highlighting significant GO terms (FDR-corrected *p* ≤ 0.05).

**REFERENCES**

**Gao, S., Yang, X., Sun, J., Zhao, X., Wang, B., and Ye, K.** (2022). IAGS: Inferring Ancestor Genome Structure under a Wide Range of Evolutionary Scenarios. Mol Biol Evol **39**.

**Garassino, F., Wijfjes, R.Y., Boesten, R., Reyes Marquez, F., Becker, F.F.M., Clapero, V., van den Hatert, I., Holmer, R., Schranz, M.E., Harbinson, J., de Ridder, D., Smit, S., and Aarts, M.G.M.** (2022). The genome sequence of *Hirschfeldia incana,* a new Brassicaceae model to improve photosynthetic light-use efficiency. Plant J. **112,** 1298-1315.

**Haas, B.J., Salzberg, S.L., Zhu, W., Pertea, M., Allen, J.E., Orvis, J., White, O., Buell, C.R., and Wortman, J.R.** (2008). Automated eukaryotic gene structure annotation using EVidenceModeler and the Program to Assemble Spliced Alignments. Genome Biol. **9,** R7.

**Holst, F., Bolger, A., Günther, C., Maß, J., Triesch, S., Kindel, F., Kiel, N., Saadat, N., Ebenhöh, O., Usadel, B., Schwacke, R., Bolger, M., Weber, A.P.M., and Denton, A.K.** (2023). Helixer–*de novo* Prediction of Primary Eukaryotic Gene Models Combining Deep Learning and a Hidden Markov Model. bioRxiv**,** 2023.2002.2006.527280.

**Joyce, B.L., Haug-Baltzell, A., Davey, S., Bomhoff, M., Schnable, J.C., and Lyons, E.** (2017). FractBias: a graphical tool for assessing fractionation bias following polyploidy. Bioinformatics **33,** 552-554.

**Nevers, Y., Rossier, V., Train, C.M., Altenhoff, A., Dessimoz, C., and Glover, N.** (2022). Multifaceted quality assessment of gene repertoire annotation with OMArk. bioRxiv**,** 2022.2011.2025.517970.

**Pham, S.K., and Pevzner, P.A.** (2010). DRIMM-Synteny: decomposing genomes into evolutionary conserved segments. Bioinformatics **26,** 2509-2516.

**Qiao, X., Li, Q., Yin, H., Qi, K., Li, L., Wang, R., Zhang, S., and Paterson, A.H.** (2019). Gene duplication and evolution in recurring polyploidization-diploidization cycles in plants. Genome Biol. **20,** 38.

**Tang, H., Bowers, J.E., Wang, X., Ming, R., Alam, M., and Paterson, A.H.** (2008). Synteny and collinearity in plant genomes. Science **320,** 486-488.
